# Comprehensive machine learning-generated classifier identifies pro-metastatic characteristics and predicts individual treatment in pancreatic cancer: A multicenter cohort study based on super-enhancer profiling

**DOI:** 10.7150/thno.84978

**Published:** 2023-05-27

**Authors:** Dongjie Chen, Yizhi Cao, Haoyu Tang, Longjun Zang, Na Yao, Youwei Zhu, Yongsheng Jiang, Shuyu Zhai, Yihao Liu, Minmin Shi, Shulin Zhao, Weishen Wang, Chenlei Wen, Chenghong Peng, Hao Chen, Xiaxing Deng, Lingxi Jiang, Baiyong Shen

**Affiliations:** 1 Department of General Surgery, Pancreatic Disease Center, Ruijin Hospital, Shanghai Jiao Tong University School of Medicine, Shanghai, P.R. China.; 2 Research Institute of Pancreatic Diseases, Shanghai Key Laboratory of Translational Research for Pancreatic Neoplasms, Shanghai Jiao Tong University School of Medicine, Shanghai, P.R. China.; 3 State Key Laboratory of Oncogenes and Related Genes, Institute of Translational Medicine, Shanghai Jiao Tong University, Shanghai, P.R. China.; 4 Department of General Surgery, Taiyuan Central Hospital, Shanxi, P.R. China.

**Keywords:** super-enhancer, machine-learning algorithm, personalized treatment, multicenter study, metastatic pancreatic cancer

## Abstract

**Rationale**: Accumulating evidence illustrated that the reprogramming of the super-enhancers (SEs) landscape could promote the acquisition of metastatic features in pancreatic cancer (PC). Given the anatomy-based TNM staging is limited by the heterogeneous clinical outcomes in treatment, it is of great clinical significance to tailor individual stratification and to develop alternative therapeutic strategies for metastatic PC patients based on SEs.

**Methods**: In our study, ChIP-Seq analysis for H3K27ac was performed in primary pancreatic tumors (PTs) and hepatic metastases (HMs). Bootstrapping and univariate Cox analysis were implemented to screen prognostic HM-acquired, SE-associated genes (HM-SE genes). Then, based on 1705 PC patients from 14 multicenter cohorts, 188 machine-learning (ML) algorithm integrations were utilized to develop a comprehensive super-enhancer-related metastatic (SEMet) classifier.

**Results**: We established a novel SEMet classifier based on 38 prognostic HM-SE genes. Compared to other clinical traits and 33 published signatures, the SEMet classifier possessed robust and powerful performance in predicting prognosis. In addition, patients in the SEMet^low^ subgroup owned dismal survival rates, more frequent genomic alterations, and more activated cancer immunity cycle as well as better benefits in immunotherapy. Remarkably, there existed a tight correlation between the SEMet^low^ subgroup and metastatic phenotypes of PC. Among 18 SEMet genes, we demonstrated that E2F7 may promote PC metastasis through the upregulation of TGM2 and DKK1. Finally, after in silico screening of potential compounds targeted SEMet classifier, results revealed that flumethasone could enhance the sensitivity of metastatic PC to routine gemcitabine chemotherapy.

**Conclusion**: Overall, our study provided new insights into personalized treatment approaches in the clinical management of metastatic PC patients.

## Introduction

Metastasis is a major cause of mortality and morbidity, which poses a great threat to the clinical management of cancer, especially in pancreatic cancer (PC). Most PC patients followed with the systemic disease at the time of diagnosis, and only 8% of patients survived more than five years after diagnosis 1. Moreover, 50% of newly identified PC patients tend to be diagnosed with metastasis, of which liver metastasis ranks first as the leading cause of death 2. Although surgical resection of the primary tumor remains the most effective strategy for prolonging patient survival, 85-90% of patients are incurable due to the systemic nature of the disease and the failure to detect the disease at an early stage 3, 4. Over the past decades, immunotherapy and molecular target therapy have revolutionized the therapeutic outcomes in most solid tumors. Unfortunately, due to the high heterogeneity and complicated immune microenvironment, few metastatic PC patients could benefit from these novel therapies 5, 6. Thus, in the era of personalized treatment, early intervention of “pro-metastatic” PC patients and identification of sensitive drugs is imperative.

Super-enhancers (SEs) are exceptionally huge clusters of enhancers that have been reported in multiple cell types 7, 8. Compared to typical enhancers, SEs collectively bind a larger number of transcription factors to facilitate the transcription of many target genes. H3K27ac (acetylation of the lysine residue at N-terminal position 27 of the histone H3 protein) is correlated with higher transcription activation and is therefore characterized as one of the frequently-used indicators for SEs 9. Research on super-enhancers has provided insights into the mechanisms that regulate gene expression and cell identity, and therefore the screening and identification of hub genes driven by SEs have suggested new strategies for the exploration of underlying biological processes 10, 11. Otherwise, elevated SE activities are reported to be involved in the metastasis of multiple cancers. SE was found to recruit transcription factors FOXA2 and HNF1A and upregulated the liver-specific gene transcription, thereby driving colorectal cancer (CRC) liver metastasis 12. By hijacking super-enhancers and subverting anti-tumor immunity, BAF155 methylation drives metastasis in triple-negative breast cancer 13. Dong J 14 also revealed that disrupting SEs by BET inhibitors is an effective approach to suppress the growth and metastasis of human head and neck squamous cell carcinoma (HNSCC) by eliminating cancer stem cells. Through SEs of ENO2 and SRC, METTL14 was demonstrated to drive metastasis and glycolytic reprogramming in the renal cell carcinoma (RCC) 15. Notably, reprogramming of the enhancer landscape was also proved to promote the acquisition of metastatic traits in PC 16, 17, but solid evidence validating the association between PC metastasis and SEs is lacking. With the help of multiple powerful machine-learning (ML) algorithms and multicenter cohorts, we can accurately characterize cancer metastasis and identify pro-metastasis modules at the resolution of SE level to better investigate the impacts of metastasis on PC.

In this study, based on 38 prognostic hepatic metastasis-acquired, super-enhancer-related (HM-SE) genes derived from SE profilings through ChIP-Seq analysis of primary pancreatic tumors (PTs) and hepatic metastases (HMs), we developed a super-enhancer-related metastatic (SEMet) classifier via 188 ML algorithm integrations in multicenter datasets. The prognostic and predictive value of the SEMet classifier was further explored and validated through a comprehensive analysis of 1 training cohort, 10 testing cohorts and 3 validation cohorts. Subgroup analysis demonstrated that the SEMet^low^ subgroup characterized the “pro-metastasis” status in PC. Additionally, the functional analysis demonstrated the correlation among 3 hub SEMet genes (DKK1, TGM2 and E2F7). At last, we screened out and confirmed that flumethasone might enhance the efficacy of gemcitabine in the treatment of metastatic PC, which would shed light on the novel personalized strategies for PC patients with metastasis. The overall workflow of our study is shown in Graphical Abstract.

## Materials and methods

### Sampling preparation and organoid establishment

For identifying the HM-SE genes, 2 patients with PT and 2 patients with HM were enrolled for H3K27ac ChIP-Seq analysis (Table S1). The samples were obtained from Ruijin Hospital, Shanghai Jiaotong University School of Medicine. The study protocol was approved by the Research Ethics Committee of Ruijin Hospital, School of Medicine, Shanghai Jiao Tong University. All enrolled participants consented to attend this cohort study and signed written informed consent. To create the metastatic patient-derived organoids (MDO), the fresh ccPDAC tissues were enzymatically digested and then treated for 1 hour at 37°C with 200 U/ml of deoxyribonuclease I (Roche) and collagenase type IV (SigmaAldrich) (Table S1). The cells plated in Basement Membrane (OuMel, #WM-MG-01) were filtered using a 70-m nylon mesh and and cultured by DMEM/F12 media containing 2% B27, N-acetyl-l-cysteine (SigmaAldrich, 1.25 mM), EGF (Gibco, 50 ng/mL), A83-01 (SigmaAldrich, 200 nM), Noggin (SigmaAldrich, 100 ng/mL), R-spondin 1 (SigmaAldrich, 500 ng/mL), Y-27632 (MedChemExpress, 10 mM), and dihydrotestosterone (SigmaAldrich, 1 nM).

### Cell culture and transfection

Pan02 cells (a murine pancreatic adenocarcinoma cell line) and human PC cell lines (Capan-2, CFPAC, Patu 8988t) were purchased from American Type Culture Collection (ATCC). The cells were cultured at 37°C in an atmosphere of 5% CO2 and respectively maintained in IMDM or DMEM medium supplemented with 10-15% FBS with 100 U/mL penicillin and 100 μg/mL streptomycin. Transient transfections were performed using lipofectamine 3000 (Invitrogen) following the instructions from ATCC guidelines.

### Chromatin immunoprecipitation (ChIP) and ChIP-qPCR experiments

The following three antibodies were used for ChIP experiments: H3K27ac (Abcam, #AB4729), E2F7 Polyclonal Antibody (ThermoFisher Scientific, #A303-037A-T) and Rabbit Control IgG (Abclonal, #AC005). ChIP assay was performed with 2 × 10^7^ adherent cells lysed to prepare nuclear extracts. Firstly, cells were treated with 1% formaldehyde to crosslink DNA. After chromatin shearing by sonication, the nuclear lysates were treated at 4°C overnight with protein A Dynabeads (Invitrogen, USA) combined with 3-5 g of antibody to prepare each sample. The beads were then retrieved with a magnet and cleaned. The DNA was then decrosslinked for 4 hours at 55°C, and purified by QIAquick PCR Purification Kit (QIAGEN, USA). 5-10 ng of pure ChIP DNA was utilized as input material for subsequent detection in each sample. At last, DNA collected from the experiments was examined by qRT-PCR assays.

### SE analysis

ROSE (Rank Ordering of Super-Enhancers) 18 was utilized to define the calling of SE with parameters (stitching distance=12.5 kb, TSS exclusive zone= +/- 2 kb) based on the ChIP-Seq peaks for H3K27ac according to a proximity rule as described in 10, 11. Then, we implemented GREAT (version 3.0.0) online tool to assign regulatory elements identified in ChIP-Seq to their putative target genes based on the association rule: basal plus extension, proximal 1kb upstream and 1kb downstream, plus distal up to 1000kb.

### RNA-seq data collection and processing

Totally, 1711 PC patients from 15 independent public cohorts were retracted from The Cancer Genome Atlas (TCGA, http://portal.gdc.cancer.gov/), International Cancer Genome Consortium (ICGC, http://dcc.icgc.org/), ArrayExpress (https://www.ebi.ac.uk/arrayexpress/), Clinical Proteomic Tumor Analysis Consortium (CPTAC, https://cptac-data-portal.georgetown.edu/) and Gene Expression Omnibus (GEO, https://www.ncbi.nlm.nih.gov/geo/). Among them, ICGC-AU-Array (n=267), TCGA-PAAD (n=176), ICGC-AU-Seq (n=81), ICGC-CA-Seq (n=182), E-MTAB-6134 (n=288), GSE62452 (n=65), GSE28735 (n=42), GSE78229 (n=49), GSE79668 (n=51), GSE85916 (n=79) and CPTAC-PDAC (n=135) with complete survival data were obtained for the establishment and validation of our classifier. Three cohorts, GSE21501 (n=102), GSE57495 (n=63) and GSE71729 (n=125) comprising entire OS information were selected as external validation cohorts. In addition, GSE151580, containing 6 paired PTs, HMs and tumor-adjacent normal pancreatic tissues (Ns), was also collected. The Fragments Per Kilobase of exon model per Million mapped fragments (FPKM) data of RNA-Seq in TCGA was downloaded from the UCSC Xena portal (https://xenabrowser.net/datapages/) and transformed into log2(TPM+1) format. The normalized expression profile of ICGC, ArrayExpress, CPTAC and GEO were downloaded from their portal. Otherwise, by removing the batch effects via the *sva* R package, Meta-cohort (n=1295) was combined from ICGC-AU-Array, TCGA-PAAD, ICGC-AU-Seq, ICGC-CA-Seq, E-MTAB-6134, GSE62452, GSE28735, GSE78229, GSE79668, GSE85916 and CPTAC-PDAC datasets. The z-score normalization of the expression matrix was applied across all datasets. The detailed clinical information of the enrolled 15 datasets was listed in Table S2.

### SEMet classifier generated from integrative machine learning algorithms

To establish a comprehensive prognosis classifier in PC, we combined 10 ML algorithms and generated 188 integrations based on those prognostic HM-SE genes. These enrolled algorithms incorporated random survival forest (RSF), elastic network (Enet), Lasso (Enet alpha=1), Ridge (Enet alpha=0), stepwise Cox, CoxBoost, partial least squares regression for Cox (plsRcox), supervised principal components (SuperPC), generalized boosted regression modeling (GBM), and survival support vector machine (survival-SVM). Then, 188 algorithm integrations were implemented to fit prediction classifiers based on 10-fold cross-validation in the ICGC-AU-Array training cohort. Finally, 10 testing cohorts (TCGA-PAAD, ICGC-AU-Seq, ICGC-CA-Seq, E-MTAB-6134, GSE62452, GSE28735, GSE78229, GSE79668, GSE85916 and CPTAC-PDAC) were applied to calculate the concordance index (C-index) value and the classifier possessed highest average C-index was deemed as optimal SEMet classifier. Detailed information is provided in Supplementary Methods.

To validate the prognostic and predictive value of the SEMet classifier, we categorized PC patients into SEMet^high^ and SEMet^low^ subgroups according to the median scores which were calculated by the optimal algorithm integration mentioned above. Kaplan-Meier curve and multivariate Cox regression analysis were performed to evaluate the prognostic value of the SEMet classifier. Otherwise, we utilized the calibration plot and receiver-operator characteristic (ROC) to appraise the predictive performance of the SEMet classifier.

### Tumor immune microenvironment (TIME) evaluation and response to immunotherapy

Gene set variation analysis (GSVA) score was calculated based on TIME signatures established by Kobayashi 19 and Bagaev 20 to assess the differences in TIME between SEMet^high^ and SEMet^low^ subgroups. The cancer immunity cycle was also measured by GSVA analysis 21, 22. Several immunotherapy predictors, including the interferon γ (IFN-γ) 23, immunophenoscore (IPS) and the Tumor Immune Dysfunction and Exclusion (TIDE) 24 score were obtained to predict response to immune checkpoint blockages (ICBs). On the other hand, the subclass mapping algorithm was performed to evaluate the expression similarity between SEMet^high^ and SEMet^low^ subgroups, and speculate the immunotherapy efficacy of anti-PD-1 and anti-CTLA-4 25.

### Genomic alteration landscape

To delve into the genomic characteristics between SEMet^high^ and SEMet^low^ subgroups, we implemented an extensive investigation in somatic mutation and copy number alteration (CNA) data in the TCGA-PAAD cohort. The *maftools* R package was employed to exhibit the mutation frequencies of the top 15 genes and we evaluated the mutational signatures through the R package *deconstructSigs* with parameters by default. Four mutational signatures showed a significant correlation with PC were inferred, namely, mutational signature 1 (age-related), mutational signature 2 (APOBEC activity-related), mutational signature 6 (DNA MMR-related) and mutational signature 15 26. Additionally, recurrent focal somatic CNAs were detected and localized by GISTIC2.0 through GenePattern (https://www.genepattern.org/), with the thresholds of copy number amplifications/deletions being equal to ±0.3 (q-value < 0.05). Regions with CNA frequency > 20% were acquired for visualization. For detecting the methylation-driven events, we adhered to the pipeline launched by Liu Z 27 and screened out key methylation-driven genes (MDGs) for PC patients. Then, we analyzed the discrepancies in methylation level and mRNA expression level between SEMet^high^ and SEMet^low^ subgroups, and further assessed the correlation between distinct methylation status and prognosis in the two subgroups.

### Annotation of metastasis-related characteristics for SEMet

To explore the association between biological characteristics and our SEMet score, we first determined significant metastatic and metabolic pathways from literature 28, 29. Then, we calculated the NES values (GSVA) of those vital pathways, and Spearman coefficients were computed to measure the similarity between those pathways and the SEMet score. Human Cancer Metastasis Database (HCMDB, http://hcmdb.i-sanger.com/index), a database developed to archive metastatic data in pan-cancer, was applied to explore the difference in expression of metastasis-related genes of PC between SEMet^high^ and SEMet^low^ subgroups. The ROC curve was plotted to validate the predictive performance of the SEMet score in distinguishing advanced tumors from early-stage tumors.

### Identification of the potential compounds

Drug sensitivity data of human cancer cell lines (CCLs) were extracted from the Cancer Therapeutics Response Portal (CTRP v.2.0, https://portals.broadinstitute.org/ctrp) and PRISM Repurposing dataset (19Q4, https://depmap.org/portal/prism/). The area under the curve (AUC) value represents the drug sensitivity in those two datasets, and lower AUC levels indicate escalated sensitivity to the treatment of distinct compounds. After imputing missing values via the K-nearest neighbor (k-NN) algorithm, compounds with more than 20% of missing data were omitted. Then, we identified the potential therapeutic compounds following the pipeline in Supplementary Methods.

### Statistical analysis

All statistical tests were performed in R software (v 4.2.2). A chi-square test was performed to compare the count data. For the measurement data that conformed to the normal distribution, the Student-t test was applied; the Wilcox test was applied for non-normal distribution data between independent subgroups. Spearman analysis was applied to estimate the correlations between two variables that are not linearly related. The Kaplan-Meier test was utilized to validate the fraction of PC patients living for a certain survival time via the *survival* package and the log-rank test was conducted to compare the significance of the difference. The *timeROC* package was used to plot the ROC curve and calibration curve. *DESeq2* package was used to call differentially expressed genes (DEGs) between two groups. Unless specifically stated, a two-tailed p-value of less than 0.05 was deemed statistically significant. See Supplementary Methods for fully detailed methods of other experiments included in our study.

## Results

### Genome-wide screening of SE and identification of HM-SE genes in PC

**Figure 1:**
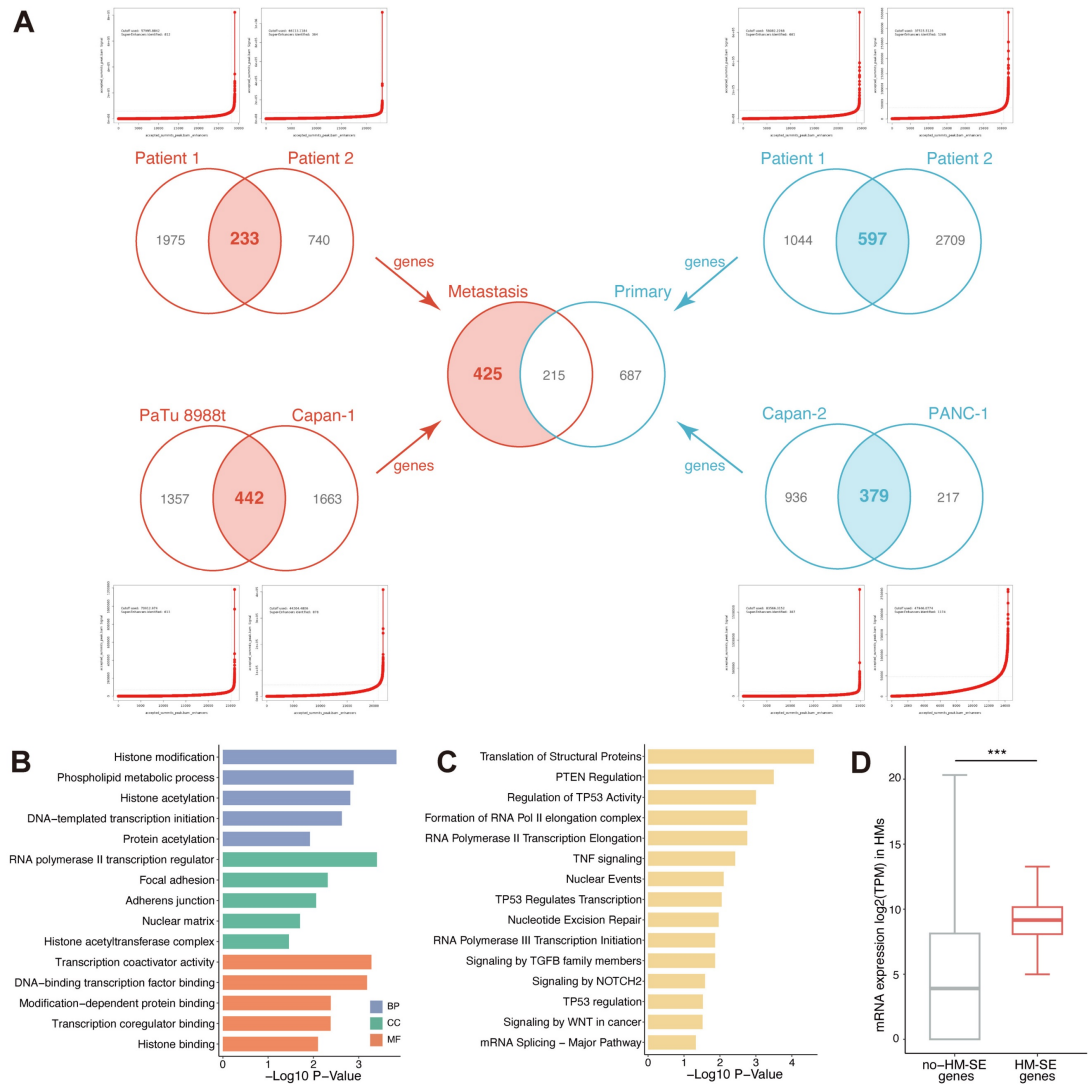
** Identification of HM-SE genes in PC**. (**A**) A total of 425 HM-SE genes were acquired from PT cell lines/samples and HM cell lines/samples in PC. (**B**) The Gene Ontology (GO) enrichment analysis demonstrated that 425 HM-SE genes were significantly associated with transcriptional regulations. (**C**) The Kyoto Encyclopedia of Genes and Genomes (KEGG) enrichment analysis illustrated that HM-SE genes were correlated with cancer sustainability. (**D**) RNA-seq results of 6 HMs suggested that the expression of the 425 HM-SE genes was higher than other genes.

To explore the implications of SE alteration in metastatic pancreatic cancer, we compared the SE landscapes among 2 PT samples, 2 PT cell lines (Capan-2 and PANC-1), 2 HM samples and 2 HM cell lines (Capan-1 and PaTu 8988t) based on H3K27ac ChIP-Seq data. For cell lines, H3K27ac ChIP-Seq data of PANC-1 and Capan-1 were obtained from public datasets (GSE149103). According to the H3K27ac signal, enhancers localized within 12.5kb were stitched and ranked. Specific enhancers that occurred above the inflection point of the H3K27ac signal were determined as SEs, and those were then annotated with genes (SE-associated genes) across all enrolled samples and cell lines (Figure 1A, Table S4-11). As is shown in Figure 1A, we converged the SE-associated genes in 2 PT samples, 2 PT cell lines, 2 HM samples and 2 HM cell lines and acquired 233, 442, 597 and 379 SE-associated genes, respectively. To systematically investigate the alterations of SE landscape during metastasis in PC, we compared the common SE-associated genes between PT and HM by merging the SE-associated genes obtained from 2 PT samples/cell lines and 2 HM samples/cell lines separately. A total of 425 HM-SE genes were identified. Then, we first characterized the functions of these HM-SE genes. Pathway enrichment analysis revealed significant enrichment in transcriptional regulation (Figure 1B) and multiple biological mechanisms that are vital for cancer sustainability (Figure 1C). We further inspected the expression of the 425 HM-SE genes in HMs, the result showed that the expression level of these genes was considerably higher than those of other genes (Figure 1D). Our results revealed that the HM-SE genes not only correlated with the regulation of SE but also related to the oncogenesis of PC.

### Integrative construction of SEMet classifier

**Figure 2:**
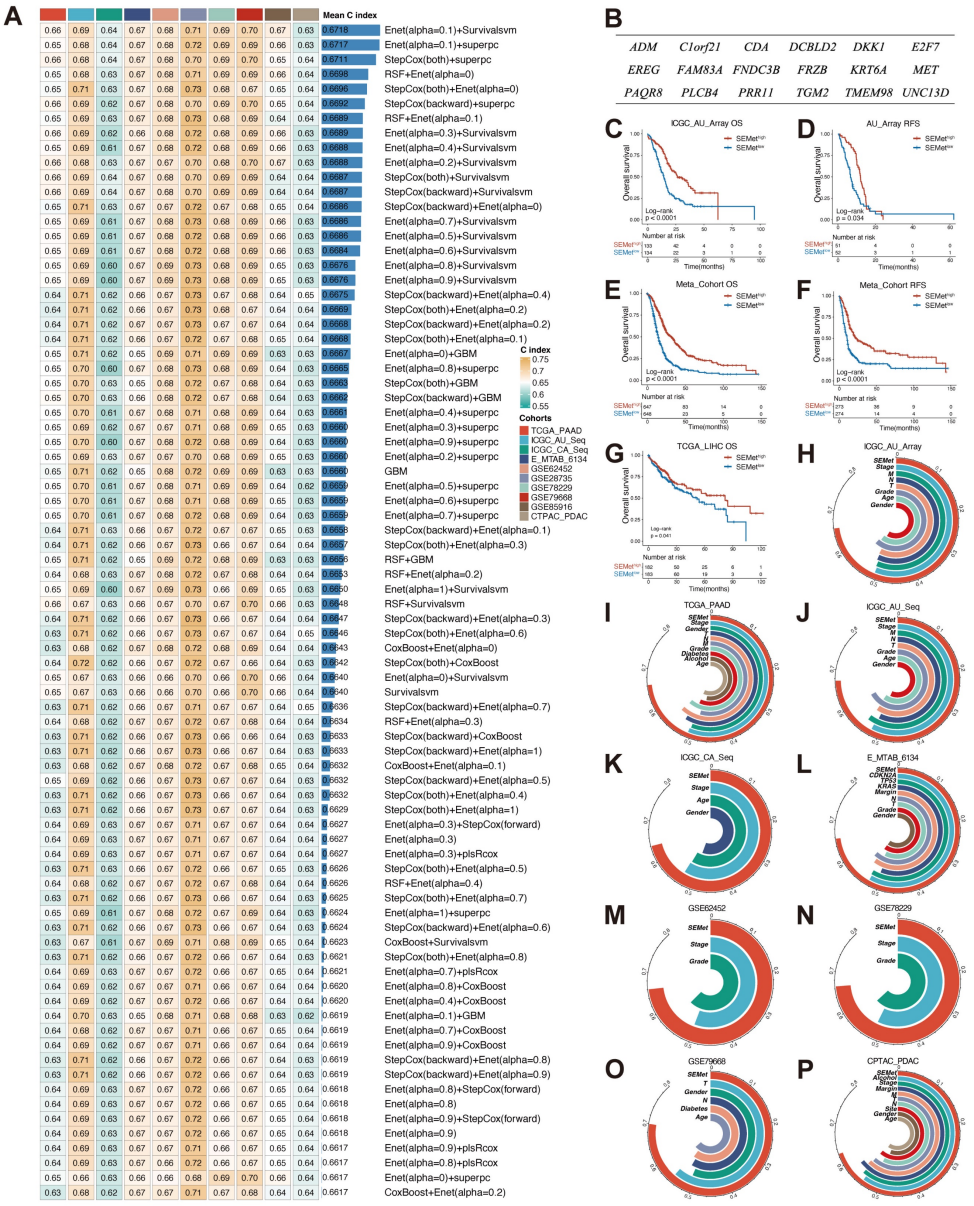
** Establishment and testing of the SEMet classifier**. (**A**) The C-indexes of the top 80 ML algorithm integrations in 10 testing cohorts. (**B**) The 18 SEMet genes. Survival analysis for OS (**C**) and RSF (**D**) between SEMet^high^ and SEMet^low^ subgroup in the ICGC-AU-Array. Survival analysis for OS (**E**) and RSF (**F**) between SEMet^high^ and SEMet^low^ subgroup in the Meta-cohort. (**G**) Kaplan-Meier curve for OS in the TCGA-LIHC. The predictive value of the SEMet classifier compared with clinical features in ICGC-AU-Array (**H**), TCGA-PAAD (**I**), ICGC-AU-Seq (**J**), ICGC-CA-Seq (**K**), E-MTAB-6134 (**L**), GSE62452 (**M**), GSE78229 (**N**), GSE79668 (**O**) and CPTAC-PDAC (**P**).

Based on the expression profile of 425 HM-SE genes, univariate Cox analysis combined with the bootstrapping method determined 38 prognostic HM-SE genes that were validated in most of the datasets in our study (Figure S1A). Next, these 38 prognostic HM-SE genes were subjected to integrative ML models to construct the SEMet classifier. ICGC-AU-Array dataset, which served as the training set, was applied to fit the 188 algorithm integrations based on 10-fold cross-validation and computed the average C-index in 10 testing datasets. The integration of Enet and survival-SVM which achieved the highest average C-index (0.6718) was identified as the optimal classifier (Figure 2A, Table S12). Moreover, the SEMet score of each sample was calculated in all 14 training and testing cohorts on the basis of 18 SEMet genes incorporated in the SEMet classifier (Figure 2B, Table S13).

### Prognostic performance of SEMet classifier

In order to assess the prognostic efficiency of the SEMet classifier, all enrolled patients were categorized into SEMet^high^ and SEMet^low^ subgroups according to the median value of the SEMet score. As is shown in Figure 2C-F, the SEMet^low^ subgroup had considerably miserable overall survival (OS) and relapse-free survival (RFS) compared with the SEMet^high^ subgroup in ICGC-AU-Array and Meta-cohort. Interestingly, in the TCGA-LIHC dataset, Kaplan-Meier curves demonstrated a similar tendency in the survival rate of HCCs (Figure 2G), suggesting that the SEMet classifier constructed in PC also has comprehensive prospects in HCC. Additionally, multivariate Cox analysis based on clinicopathological characteristics demonstrated that the SEMet classifier was an independent prognostic factor in PACA-AU-Array and Meta-cohort (all p < 0.05, Figure S2).

In the other 10 testing cohorts, the survival analysis consistently revealed a markedly prolonged OS time in the SEMet^high^ subgroup than those in the SEMet^low^ subgroup (all p < 0.05, Figure S3). Likewise, the Kaplan-Meier analysis also demonstrated that patients in the SEMet^low^ subgroup had an unfavorable RFS in TCGA-PAAD, ICGC-CA-Seq and E-MTAB-6134 datasets (all p < 0.05, Figure S3). Similarly, after adapting for obtained clinicopathologic information, multivariate Cox analysis confirmed that the SEMet classifier was a protective factor for OS (all p < 0.05, Figure S2). Consistently, the SEMet classifier remained statistically significant for RSF in TCGA-PAAD, ICGC-CA-Seq and E-MTAB-613 cohorts (all p < 0.05, Figure S2). Given the results mentioned above, our SEMet classifier holds a robust level of prognostic performance and was an independent prognostic factor in PC.

### Predictive value of SEMet classifier

**Figure 3:**
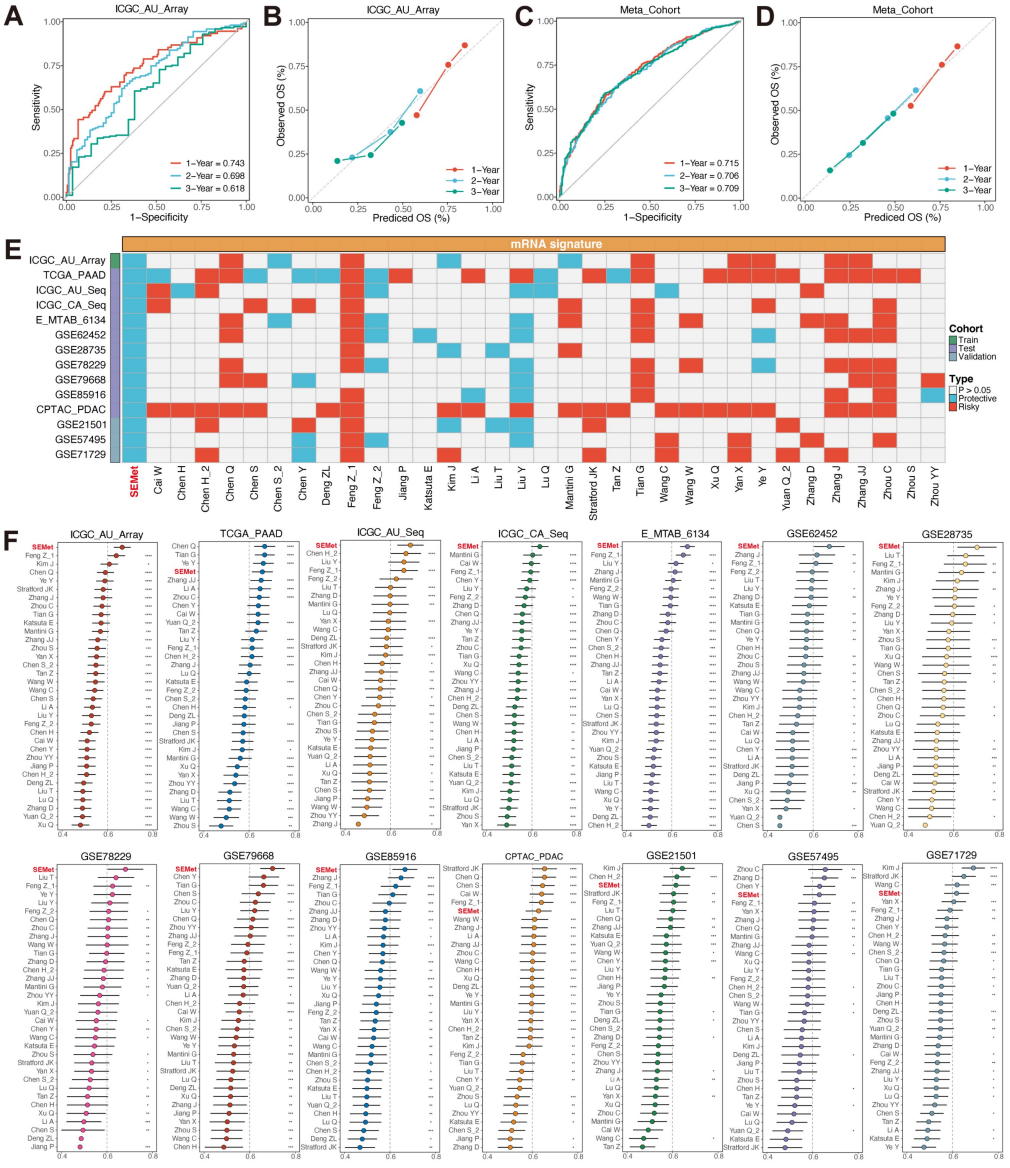
** Predictive performance of SEMet classifier**. Calibration plot for predicting 1-, 2- and 3-year OS in ICGC-AU-Array (**A**) and Meta-cohort (**C**). ROC curve for predicting 1-, 2- and 3-year OS in ICGC-AU-Array (**B**) and Meta-cohort (**D**). (**E**) Univariate Cox analysis of SEMet classifier and 33 published signatures of PC. (**F**) Comparison of C-index and 33 published signatures in ICGC-AU-Array, TCGA-PAAD, ICGC-AU-Seq, ICGC-CA-Seq, E-MTAB-6134, GSE62452, GSE28735, GSE78229, GSE79668, GSE85916, CPTAC-PDAC, GSE21501, GSE57495 and GSE71729.

ROC curves were plotted to evaluate the sensitivity in the prediction of the SEMet classifier (Figure 3A, C and Figure S4). The calibration plots of the ICGC-AU-Array training set, Meta-cohort and 10 testing cohorts also confirmed that the SEMet classifier obtained an excellent value of prediction (Figure 3B, D and Figure S5). All these proofs exhibited that the SEMet classifier had a certain efficiency in multicenter cohorts. Numerous studies have confirmed that clinical traits and genetic alterations predict the prognosis of PC. Hence, we compared the predicted efficacy of the SEMet classifier with other clinical variables in 9 cohorts with completed clinical information. As is shown in Figure 2H-P, the C-index of the SEMet classifier had superior accuracy than other variables. Additionally, to further validate the classifier more scrupulously, we assessed the predictive capacity of the SEMet classifier in 3 validation datasets. Patients in the SEMet^low^ subgroup owned decreased survival rates in GSE21501, GSE57495 and GSE71729 cohorts (all p < 0.05, Figure S3). The AUC and calibration curve also determined the consistent predictive performance of the SEMet classifier (Figure S4-5). In summary, according to survival analysis, multivariate Cox analysis, ROC curve, calibration plot and C-index comparison, our SEMet classifier achieved sufficient to excellent performance in 1 training cohort, 10 testing cohorts and 3 validation cohorts. These results led us to deduce that the SEMet classifier may stress its potential as a predictive tool in the clinical management of PC.

### Comparison of multiple prognostic signatures in PC

The development of next-generation sequencing witnessed the construction of extensive prognostic signatures and classifiers based on various ML algorithms. We compared the performance of the SEMet classifier with 33 previous well-established predictive gene signatures related to a variety of biological characteristics such as immune microenvironment, ferroptosis, hypoxia, etc (Table S14). First of all, we conducted the univariate Cox analysis, and only our SEMet classifier had consistent significance across 14 independent cohorts (Figure 3E). Then, the C-index was also calculated to compare with all enrolled signatures. SEMet classifier revealed superior efficiency in each dataset than almost all signatures (ranked first in 9/14 datasets, Figure 3F). Notably, most of the signatures achieved higher stability in their training dataset but performed far from satisfactory in other external cohorts, which may be thanks to the poor generalisability and overfitting. Via integration and permutations of ML algorithms, our final optimal classifier was proved to reduce redundancy significantly and performed well.

### Immune characteristics and immune response prediction of SEMet classifier

**Figure 4:**
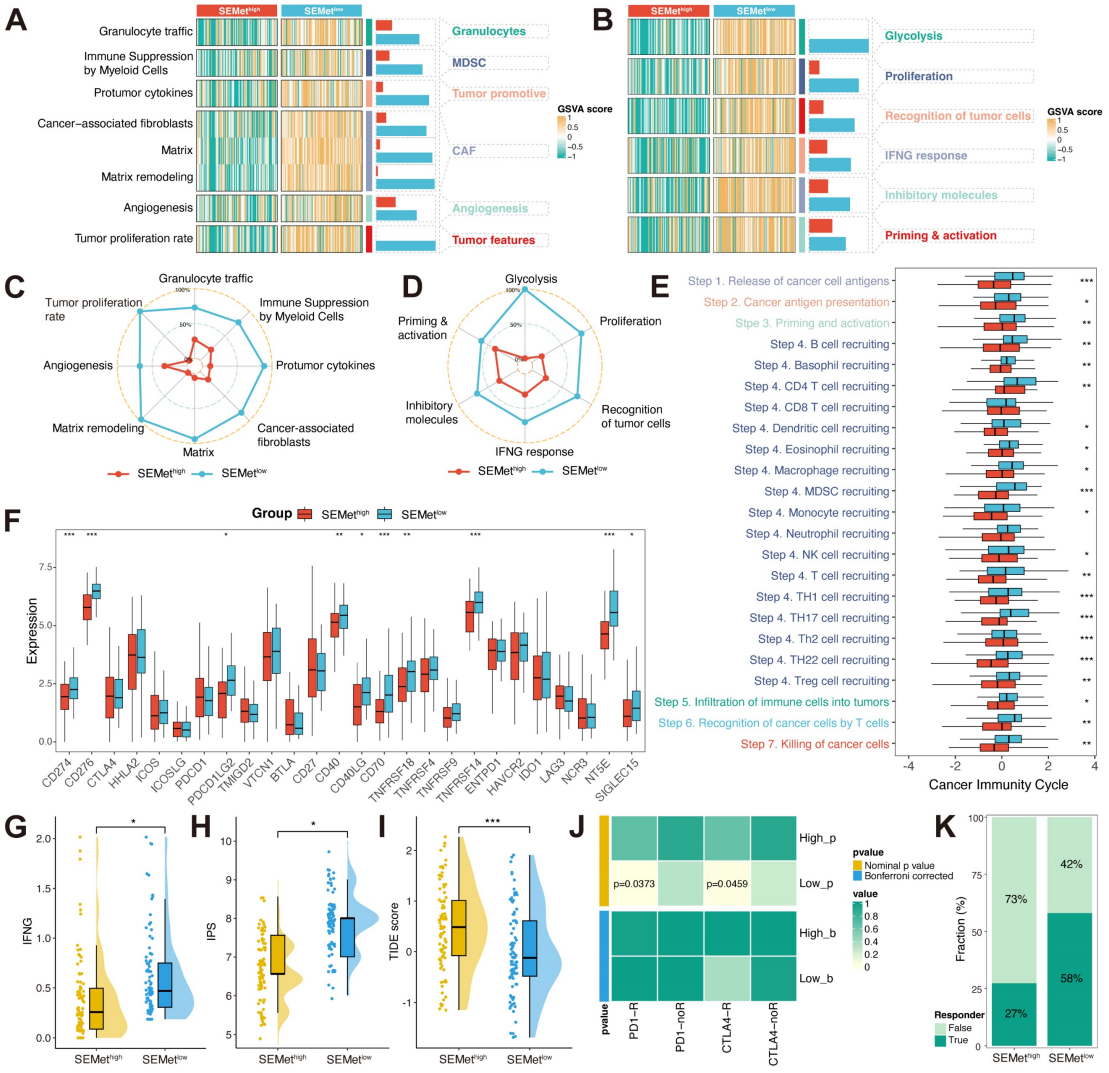
** The immune landscape between SEMet^high^ and SEMet^low^ subgroup**. Heatmap of GSVA score based on Kobayashi (**A**) and Bagaev (**B**) TIME signatures. The radar chart revealed the differences in TIME signatures developed by Kobayashi (**C**) and Bagaev (**D**) between SEMet^high^ and SEMet^low^ subgroups. (**E**) Boxplot displayed the cancer immunity cycle differences between SEMet^high^ and SEMet^low^ subgroups. (**F**) Boxplot of expression of 27 ICBs between SEMet^high^ and SEMet^low^ subgroups. Boxplot illustrated the IFN-γ (**G**), IPS (**H**) and TIDE score (**I**) between SEMet^high^ and SEMet^low^ subgroups. (**J**) Contingency table between immunotherapy responses (anti-PD-1 and anti-CTLA-4) and SEMet groups based on SubMap analysis. (**K**) Percentage of immune responses between SEMet^high^ and SEMet^low^ subgroups.

TIME is confirmed to play an indispensable role in metastatic PC by molding pre-metastatic sites into an immunosuppressive environment. According to two TIME signatures, the SEMet^low^ subgroup was associated with higher levels of MDSCs, CAFs, granulocytes, angiogenesis, tumor-related features by Bagaev (Figure 4A, C) and glycolysis, proliferation, recognition of tumor cells, INF-γ response, inhibitory molecules, priming & activation by Kobayashi (Figure 4B, D) in TCGA-PAAD dataset. To further detect the character of the SEMet classifier in response to immunotherapy, we first calculated the cancer immunity cycle which represents the state of checkpoints and inhibitors in the immune response. Extraordinary, most steps of the cancer immunity cycle were more activated in the SEMet^low^ subgroup in the TCGA-PAAD dataset (Figure 4E). In addition, the SEMet^low^ subgroup had higher expression levels of ICBs (Figure 4F). Furthermore, the association between the SEMet classifier and immunotherapy indicators was explored. A lower SEMet score was more correlated with a higher IFN-γ level (Figure 4G), a higher IPS score (Figure 4H) and a lower TIDE score (Figure 4I), all of which were predictors of superior immunotherapy response. Besides, the Submap algorithm confirmed that the SEMet^low^ subgroup tended to benefit from immunotherapy responses (Figure 4J-K).

### Genomic landscape of SEMet classifier

**Figure 5:**
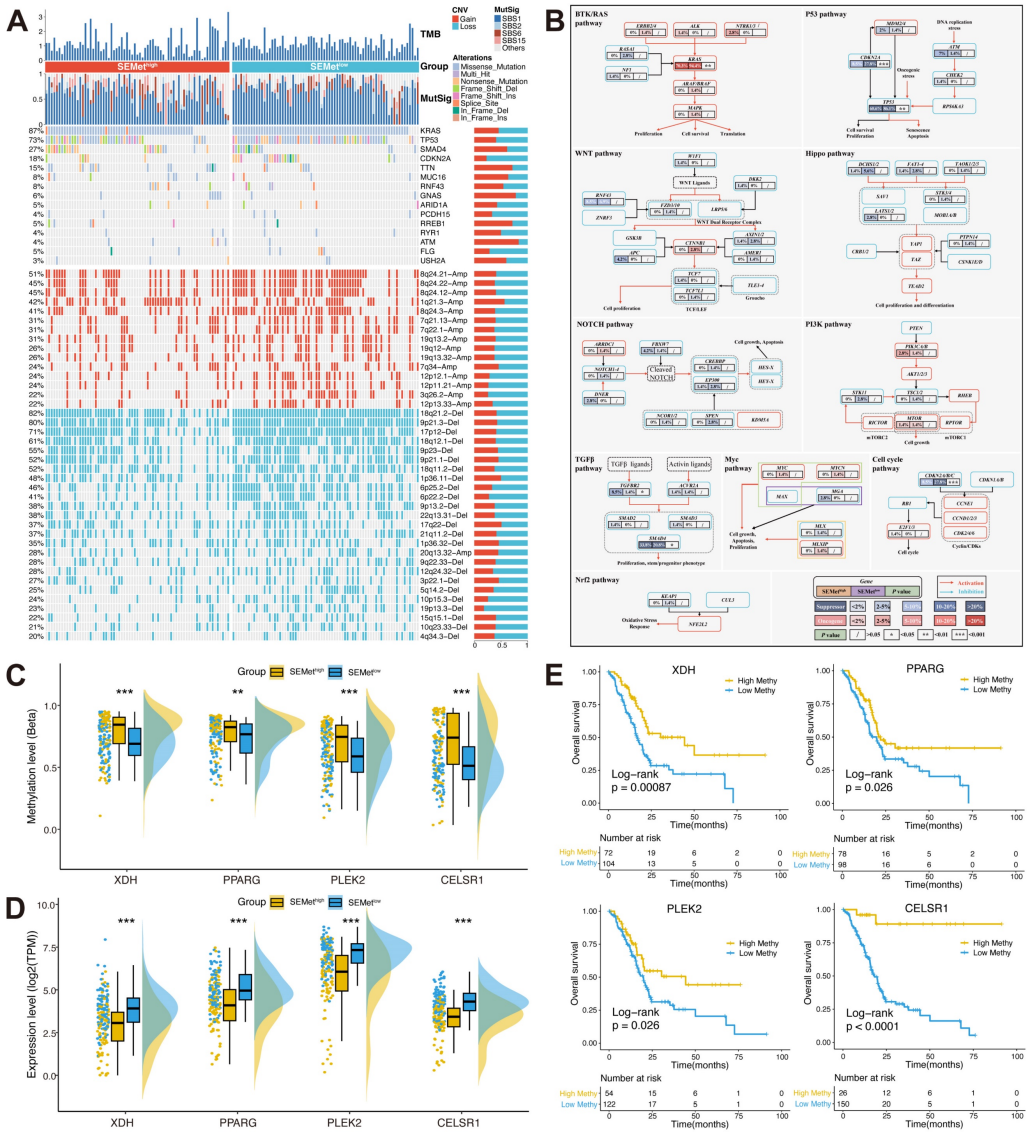
** Muti-omics analysis based on mutation, CNV and methylation**. (**A**) Comparison of somatic mutation and CNV between SEMet^high^ and SEMet^low^ subgroups. (**B**) Mutation landscape in 10 canonical oncogenic pathways between SEMet^high^ and SEMet^low^ subgroups. Boxplot of methylation level (**C**) and expression of 4 MDGs (**D**). (**E**) Survival analysis between high and low methylation groups in 4 MDGs.

To inspect the genomic landscape between SEMet^high^ and SEMet^low^ subgroups, we implemented a broad analysis of somatic mutations and CNA. As is shown in Figure 5A, the SEMet^low^ subgroup exhibited a higher tumor mutation burden (TMB) level than the SEMet^high^ subgroup. In the analysis of mutation signatures in PC, we found that mutation signature 1 (age-related) was abundant in the SEMet^low^ subgroup. In contrast, mutation signature 6 (DNA MMR-related) was enriched in the SEMet^high^ subgroup. Accumulating evidence has demonstrated that the tumor oncogene/suppressor gene mutation plays a critical role in initiating and maintaining PC and its related signaling network. Herein, we further assessed the mutation rate of genes in 10 classical oncogenic pathways in TCGA 30. Of note, the SEMet^low^ subgroup possessed significant mutation of tumor suppressor genes (TP53 and CDKN2A) and oncogene (KRAS), while the SEMet^high^ subgroup did the opposite (Figure 5B). Additionally, we explored the CNV characteristics between two subgroups. Compared to the SEMet^high^ subgroup, the SEMet^low^ subgroup obtained higher amplification of 8q24.21 (oncogene MYC located), 8q24.22, 8q24.12 and deletion of 18q21.2 (suppressor gene CDKN2A/B located), 9p21.3 (suppressor gene SMAD4 located), 17p12. Overall, we could hypothesize that the alterations of tumor oncogene/suppressor gene in the genomic landscape lead to the distinct features between SEMet^high^ and SEMet^low^ subgroups.

Furthermore, we investigated the MDGs to unveil the methylation-driven events in PC based on our SEMet classifier. Results showed that four MDGs (XDH, PPARG, PLEK2 and CELSR1) gained lower methylation levels and higher mRNA expression levels in the SEMet^low^ subgroup compared to the SEMet^high^ subgroup (Figure 5C-D). In survival analysis, we concluded that the lower methylation level group achieved significantly shorter overall survival in four identified MDGs (Figure 5E), which implies methylation also plays an essential part in the SEMet classifier.

### Correlation between the SEMet classifier and published PC classifications

**Figure 6:**
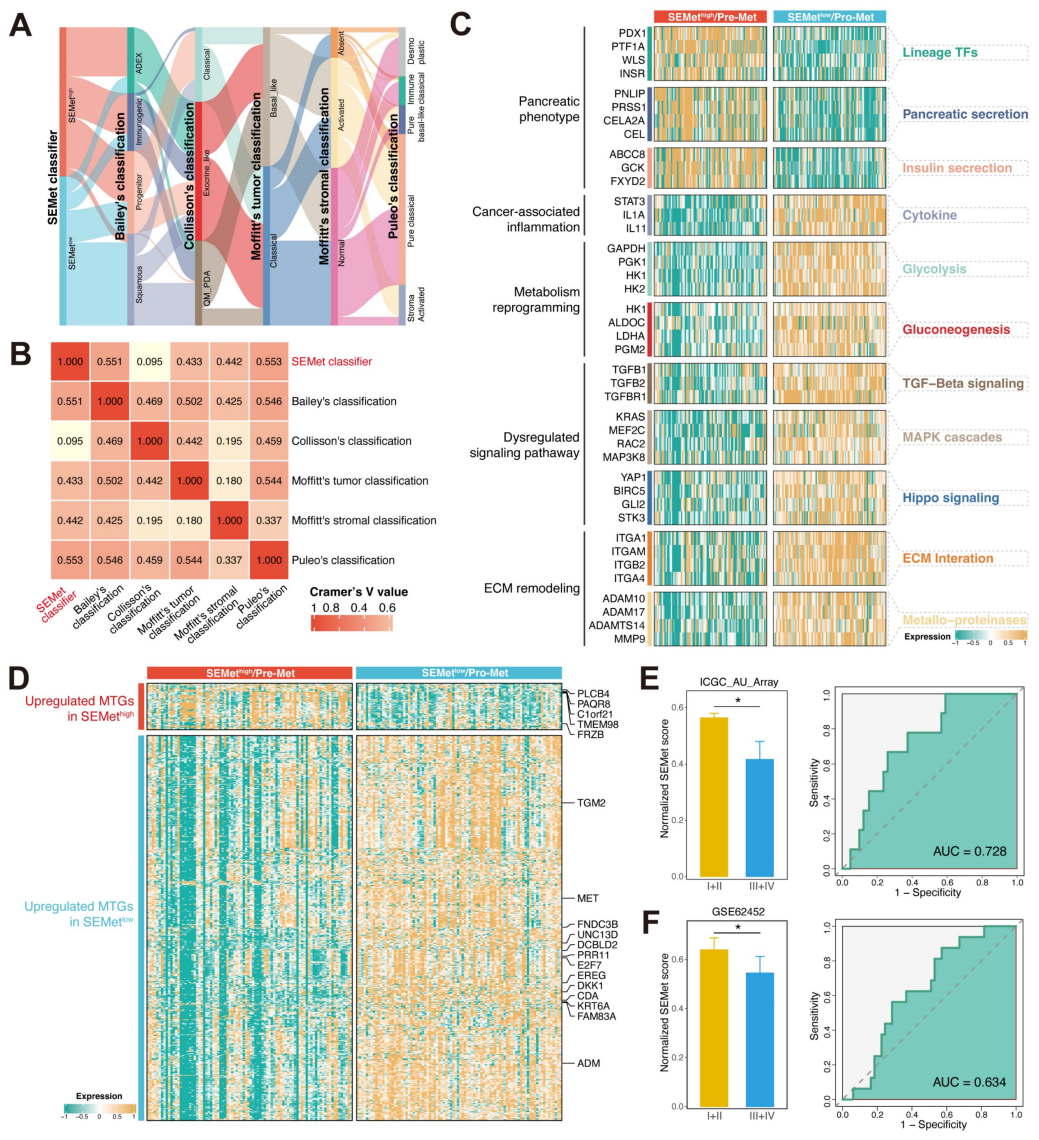
** The biological function of the SEMet^low^ subgroup**. (**A**) The alluvial plot displayed the relationship between the SEMet classifier and other molecular classifications. (**B**) Heatmap of Cramer's V statistic reflected the corrections between six PC molecular classifications. (**C**) The dynamic regulation pattern of gene expression across SEMet^high^ and SEMet^low^ subgroups based on basic molecular hallmarks. (**D**) The expression of metastasis-related genes of PC in HCMDB. ROC curve for predicting advanced tumors of SEMet classifier in ICGC-AU-Array (**E**) and GSE62452 (**F**).

Next, we compared the SEMet classifier with reported molecular subtypes in PC. The signature genes of Bailey's classification, Collisson's classification, Moffitt's tumor classification and Moffitt's stromal classification were utilized to cluster PC patients in the TCGA-PAAD cohort (Figure S6A-D, Table S15), and Puleo's classification was predicted followed the pipeline in Supplementary Methods (Table S15). Results illustrated that there was no significant difference between Collisson's classification and SEMet classifier (p = 0.45, Table S16), while Bailey's classification (p < 0.0001), Moffitt's tumor classification (p < 0.0001), Moffitt's stromal classification (p < 0.0001) and Puleo's classification (p < 0.0001) exhibited considerable correlations (Table S16). For the combination of Bailey's classification, we found that the proportion of squamous subtype was higher and the percentage of other subtypes was lower in SEMet^low^ subgroup versus SEMet^high^ subgroup (55.68% vs 5.68%, 21.59% vs 34.09%, 11.36% vs 27.27%, 11.36% vs 32.95%, p < 0.0001). With regard to Moffitt's tumor classification, we observed that SEMet^low^ subgroup was composed of a more basal-like subtype and a less classical subtype compared to SEMet^high^ subgroup (68.18% vs 25.00%, 31.82% vs 75.00%, p < 0.0001). For Moffitt's stromal classification, results demonstrated that the SEMet^low^ subgroup possessed a more activated subtype and less normal subtype than SEMet^high^ subgroup (57.95% vs 15.91%, 37.50% vs 68.18%, p < 0.0001). With respect to Puleo's classification, the frequency of desmoplastic and immune classical was lower within SEMet^low^ subgroup (6.82% vs 26.14%, 18.18% vs 1.14%, p < 0.0001). On the contrary, we also found a higher frequency of pure basal-like and stroma activated subtypes in SEMet^low^ subgroup versus SEMet^high^ subgroup (19.32% vs 0.00%, 23.86% vs 3.41%, p < 0.0001). The alluvial plot exhibited that the SEMet classifier had a robust relationship with other molecular classifications (Figure 6A). Furthermore, the similarity between the SEMet classifier and other published classifications was quantified by Cramer's V (Figure 6B). We observed that the SEMet classifier had the highest correlation with Puleo's classification (Cramer's V value = 0.553) and the lowest relationship with Collisson's classification (Cramer's V value = 0.095). In conclusion, it was demonstrated that the SEMet classifier was significantly correlated with other PC classifications and the prognosis of PC patients.

### SEMet^low^ subgroup is associated with pro-metastasis functions

Since the development of the SEMet classifier is based on the SE profile of HMs, we assumed that there were differences in biological functions related to metastasis at different levels of SEMet scores. Hence, based on basic molecular hallmarks, we studied the dynamic regulation pattern of gene expression across SEMet^high^ and SEMet^low^ subgroups. As is shown in Figure 6C, the SEMet^low^ subgroup was significantly enriched in biological processes such as cancer-associated inflammation, metabolism reprogramming, dysregulated signaling pathway and ECM remodeling, while the SEMet^high^ subgroup possessed more pancreatic phenotype. In consistency, GSEA further validated that the SEMet^low^ subgroup owned a more malignant phenotype (Figure S7A). Interestingly, we also observed that the SEMet score had a significant negative correlation with the glycolysis pathway and metabolic activity of metabolites, such as glycogen and retinoids (Figure S7B), suggesting that the SEMet^low^ subgroup adopts a distinct metabolism fashion as those in the SEMet^high^ subgroup. To further explore the metastatic features between two subgroups, we found that all of the SEMet genes were enrolled in the HCMDB dataset and most of the SEMet genes (13/18, 72%) were upregulated in the SEMet^low^ subgroup, implying that the SEMet^low^ subgroup possessed more metastatic characteristics in PC (Figure 6D). Thus, based on the above results, we deduced that the SEMet^low^ subgroup might represent the “pro-metastasis” status, and the SEMet^high^ subgroup might stand for the “pre-metastasis” environment in PC. To validate this hypothesis, subgroup analysis was performed in ICGC-AU-Array and GSE62452 datasets, and we observed that patients in the SEMet^low^ subgroup gained more advanced stages, which might result in dismal OS (Figure 6E-F). Regarding the metastasis prediction of SEMet score, AUC reached 0.728 in ICGC-AU-Array cohorts and 0.634 in GSE62452 cohorts (Figure 6E-F). While the AUC of GSE62452 was not satisfactory, it is probably due to the limited sample size.

### Prognostic performance of 18 SEMet genes

To examine the prognostic performance of 18 SEMet genes, we performed the univariate Cox analysis based on the SEMet classifier and its 18 SEMet genes in comprehensive multicenter cohorts (1 training set, 10 testing sets and 3 validation sets). As illustrated in Figure S8, the SEMet classifier was an independent protective factor across all 14 cohorts, while SEMet genes performed hardly well in several datasets. Besides, we established 19 classifiers by 10 ML algorithms based on the expression of 18 SEMet genes in ICGC-AU-Seq cohorts. Then, the classifier constructed by survival-SVM, namely our SEMet classifier, ranked first across 19 classifiers among the average C-index of 13 cohorts (Figure S1B). In other words, our SEMet classifier, which was developed by combining Enet in dimensionality reduction and survival-SVM in feature selection, was the optimal integration and performed the best prognostic value.

### DDK1 and TGM2 act in PC as a pro-metastasis factor targeted by E2F7

**Figure 7:**
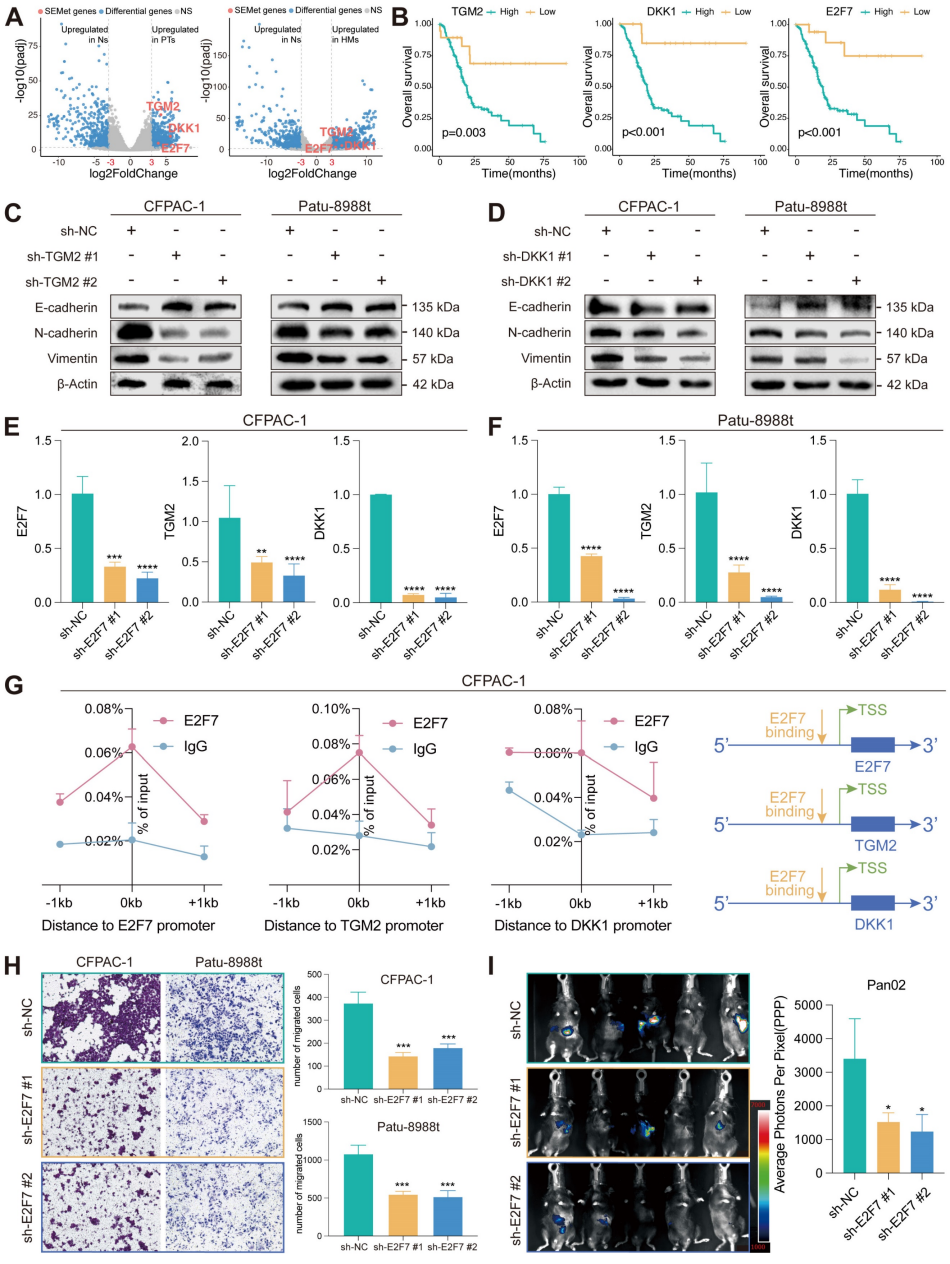
** E2F7 targets TGM2 and DKK1 to promote the proliferation of PC. (A)** Volcano plot illustrated that TGM2, DKK1 and E2F7 were the top 3 genes upregulated in PTs and HMs compared to Ns. (**B**) Survival analysis demonstrated that the higher expression of TGM2, DKK1 and E2F7 led to a dismal survival rate in the TCGA-PAAD dataset. The protein expression of EMT markers in CFPAC-1 and Patu 8988t after knockdown of TGM2 (**C**) and DKK1 (**D**). Expression of TGM2 and DKK1 in control and sh-E2F7 CFPAC-1 (**E**) and Patu 8988t (**F**). (**G**) Binding of E2F7 at the promoter regions of TGM2 and DKK1 in CFPAC-1 based on ChIP-qPCR. (**H**) Transwell assays validated the pro-metastatic efficacy of E2F7 in CFAPC-1 and Patu 8988t. (**I**) BLI demonstrated that E2F7 knockdown remarkably decreased the metastatic ability of PDAC cells into the liver compared to that of the control group.

Differential analysis revealed that three SEMet genes (TGM2, DKK1 and E2F7) were significantly upregulated in HMs and PTs compared to Ns by criteria of logFC > 3 and FDR < 0.01 (Figure 7A). In the meantime, survival analysis demonstrated that the expression of TGM2, DKK1 and E2F7 were negatively correlated with the survival rate (Figure 7B). To further confirm the efficacy of SEMet genes in pancreatic cancer metastasis, we examined the protein expression of EMT markers after TGM2 or DKK1 knockdown in two metastasis lesion-originated cell lines, CFPAC-1 and Patu 8988t. The epithelial marker, E-cadherin, was overexpressed after the knockdown of TGM2 or DKK1. On the contrary, mesenchymal markers including N-cadherin and Vimentin were down-regulated (Figure 7C-D, Figure S9). Among these three SEMet genes, E2F7 was reported to function as a transcriptional regulator that promotes cell proliferation and metastasis in multiple cancers 31-33. Therefore, we speculated that E2F7 binds to the promoter region of TGM2 and DKK1, and actively promoted them which was then demonstrated by RT-qPCR (Figure 7E-F) and E2F7 targeted ChIP-qPCR experiments (Figure 7G). Besides, *in vitro* and *in vivo* experiments were conducted to assess the impacts of E2F7 on the metastasis of PC. Transwell assays validated the pro-metastatic efficacy of E2F7 in CFAPC-1 and Patu 8988t cell lines (Figure 7H). Also, stable luciferase-labeled Pan02 PC cells, with or without E2F7 knockdown were established. Then, we injected them into the spleen vein of C57 mice. After 21 days, we observed that E2F7 knockdown remarkably decreased the metastatic capacity of PC cells into the liver compared to the control group, as measured by bioluminescence imaging (BLI) of HMs (Figure 7I). In collaboration, all results implied that E2F7 may contribute to PC metastasis by upregulating TGM2 and DKK1.

### Identification and validation of potential therapeutic compounds for metastatic PCs based on SEMet classifier

Now that the SEMet^low^ subgroup could effectively symbolize the “pro-metastasis” status in PC, comprehensive approaches were adopted to screen out candidate therapeutic compounds targeting SEMet^low^ subgroup patients. Based on the drug sensitivity data in CTRP and PRISM, the *oncopredict* R package was utilized to predict the drug response data of patients in the TCGA-PAAD cohort. Before processing further, we demonstrated that the result of predicted drug response data was reliable. PTBP3, PTB protein 3, was first identified as an essential RNA-binding protein in 1999. A recent study revealed that PTBP3 increases PC proliferation in response to hypoxic stress, contributing to gemcitabine resistance 34. We thus categorized patients into two groups according to the median expression of BTBP3. The result revealed that patients in the PTBP3_high group showed significantly higher estimated AUC values of gemcitabine, which is consistent with the previous experiment data (Figure S10A).

**Figure 8:**
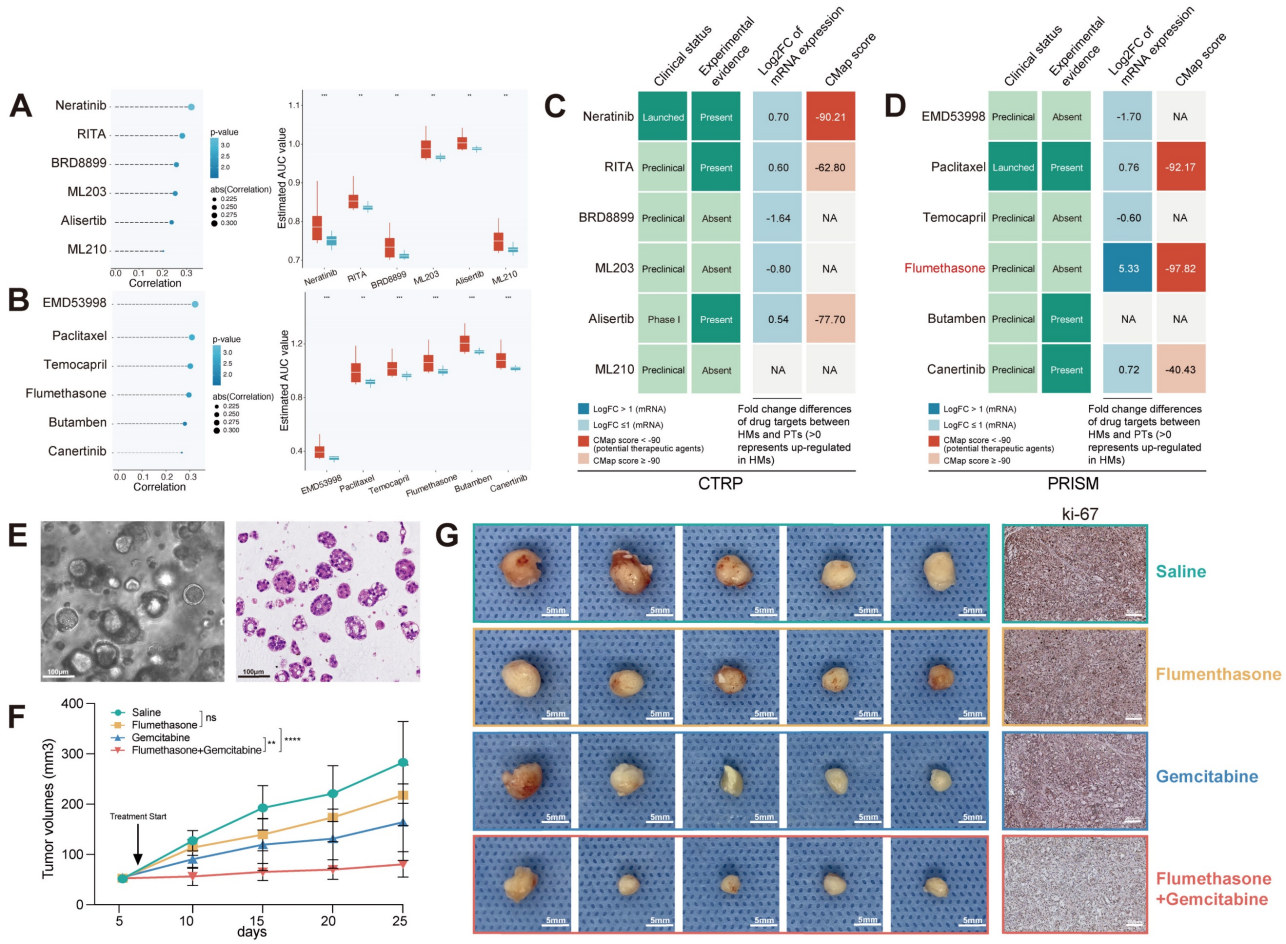
** FLM served as an adjuvant sensitizer to increase the efficacy of gemcitabine in metastatic PC.** The results of Spearman's correlation analysis and differential drug response of six CTRP-derived (**A**) and six PRISM-derived (**B**) candidate compounds. (**C-D**) Identification of the most reliable compound based on evidence from multiple sources. (**E**) Establishment of a PC MDO (scale bars, 100 µm). (**F**) The growth rate of tumors in response to the treatment of saline, gemcitabine (6mg/kg) , flumethasone (0.1mg/kg) and combined administration (n=5/group). (**G**) Representative images of subcutaneous PC tumors (scale bars, 5 mm) and Ki-67 staining (scale bars, 200 µm) of tumor tissues of mice treated with saline, gemcitabine (6mg/kg), flumethasone (0.1mg/kg) and combined administration (n=5/group).

Then two methods were conducted to identify the potential compounds via CTRP and PRISM databases (Supplementary Methods). As is shown in Figure 8A-B, we generated 6 CTRP-derived compounds (Neratinib, RITA, BRD8899, ML203, Alisertib and ML210) and 6 PRISM-derived compounds (EMD53998, Paclitaxel, Temocapril, Flumethasone, Butamben and Canertinib). Although these 12 compounds possessed a higher drug sensitivity in the SEMet^low^ subgroup, the above analyses could hardly support the conclusion that these compounds had an effective clinical application on metastatic PCs. Hence, we first search the literature in PubMed (https://www.ncbi.nlm.nih.gov/pubmed/) to discover the clinical and experimental proofs of these candidate compounds. Secondly, differences in mRNA expression of candidates' drug targets were measured by fold changes between HMs and PTs. The higher value of fold-change represented a considerable potential of candidates in treating metastatic PCs. Thirdly, the CMap analysis was performed to explore the sensitive compounds based on the differential genes between HMs and PTs (Table S17). Taking all the results into consideration, Flumethasone, which has robust evidence, may hold the most promising and novel potential in the treatment of metastatic PCs, although other routine candidates have already been in clinical trials (Figure 8C-D).

Flumethasone (FLM), a glucocorticoid with anti-inflammatory, vasoconstrictive and anti-hyperplasia properties, was merely confirmed to enhance the efficacy of chemotherapeutic management in lung cancer 35. Since the chemoresistance of gemcitabine still leads to poor clinical outcomes in metastatic PCs 36, which was also demonstrated based on our estimated drug sensitivity (Figure S10B), we hypothesized that FLM might be served as an adjuvant sensitizer to increase the efficacy of gemcitabine in metastatic PCs. Currently, patient-derived organoids are considered one of the most suitable pre-clinical models to exhibit similarity to original tumors. Thus, we established a PC MDO model (Figure 8E) to further demonstrate the efficacy of Flumethasone combined with gemcitabine in metastatic PC treatment. Firstly, the subcutaneous xenograft model was generated using MDO in Balb/c nude mice, with 2*10^6 organoid crypts for each injected tumor. Mouse were divided into four groups and were treated with saline, gemcitabine (6mg/kg), flumethasone (0.1mg/kg) 35 or combined administration (n=5/group). After 14 days, it was found that combined administration of gemcitabine and flumethasone remarkably suppressed *in vivo* MDO tumor size and ki-67 expression (Figure 8F-G, Figure S11). These data suggested the potential clinical usage of flumethasone combined with routine gemcitabine chemotherapy in metastatic or progressive PC patients.

## Discussion

Pancreatic cancer is one of the most devastating malignancies and is predicted to surpass prostate, breast, and colorectal cancers to become the second leading cause of cancer death by 2030 37. Identifying novel biomarkers and establishing stratified treatment is essential for improving the prognosis of PC, especially in metastatic disease. Over the past decades, a vast number of studies have demonstrated that dysregulated epigenetic control of cancer cells is strictly correlated with malignant transformation and metastasis 38, 39. The super-enhancer concept is significantly critical in cancer research, and the acquisition of SEs around oncogene drivers is widely observed during the process of tumorigenesis in PC 40-42. However, the biological meaning of SE in metastatic PC remains unclear, and it is useful to screen out critical SEs and associated genes that are required for the metastasis of PC.

In our study, a total of 425 HM-SE genes were identified based on the ChIP-Seq profiling among PTs and HMs. Strikingly, these genes were acquired for the formation of SE and PC progression. Based on the expression of these genes, we constructed a comprehensive guideline to establish the SEMet classifier. To sum up, 188 integrations of models were fitted to the training set via the 10-fold cross-validation. Then, based on 10 independent testing cohorts, the integration of Enet (α = 0.1) and survival-SVM was determined as the optimal model. After minimizing redundant noise by Enet, we finally obtained an 18-gene classifier termed SEMet classifier via survival-SVM. This novel computational framework to develop a consensus SEMet classifier could effectively reduce the overfitting encountered by ML algorithms.

Among the 18 SEMet genes identified for the SEMet classifier, 12 have been reported. CDA regulates the metabolism of epigenetic nucleosides revealing a therapeutic window in cancer, especially in pancreatic cancer, that has CDA overexpression and is resistant to treatment with other cytidine analogs 43, 44. Based on bioinformatics analyses, DCBLD2 was confirmed to be a considerably oncogenic factor in PC with diagnostic, prognostic and therapeutic potential 45. DKK1 could be served as an excellent target for cancer immunotherapy, and DKK1-CKAP4-PI3K/AKT signal pathway also plays a pivotal role in the proliferation of PC 46. By targeting E2F7, miR-26a inhibited the malignant behaviors of PC cells 47. In pancreatic adenosquamous carcinoma, EGFR-associated ligand-receptor pairs are activated in cell communications at a single-cell transcriptomics level 48. FAM83A regulates Wnt/β-catenin signaling by directly binding to β-catenin and suppressing TCF4-mediated transcriptional activity, leading to pancreatic tumorigenesis 49. FRZB, coding for modulators of the non-canonical WNT signaling pathway, could be sustained by adipocytes to maintain the PC progression 50. KRT6A alters the tumor-associated macrophage subtypes and indicates an undesirable prognosis in PC 51. MET is regarded as a pancreatic cancer-specific RTK, which is significantly related to prognosis in PC 52. PLCB4 correlates with the p53 status and prognosis of PC patients 53. Via inhibiting the PRR11, USP34 could promote the proliferation and migration in PANC-1 cells 54. ROS-dependent apoptosis could be induced by kaempferol via TGM2-mediated Akt/mTOR signaling in PC 55.

The prognosis and predictive value of the SEMet classifier were validated in multicenter cohorts via survival analysis, univariate Cox analysis, ROC and calibration plot. Results revealed that the SEMet classifier was a stable factor of OS and RSF in PC patients. Otherwise, 33 established signatures of vital functional gene sets were screened out for comparison based on C-index. The SEMet classifier exhibited significantly excellent performance across almost all datasets. We noticed several signatures (e.g. Stratford JK, Chen Q, etc.) performed better than the SEMet classifier in certain cohorts, but they performed weakly in most other validation cohorts, revealing their poor universality and generalizability. Our classifier was reduced redundant and selected via combing two ML algorithms and obtained an advanced predictive effect.

The TIME induced by the interaction between PC cells and stromal cells is essential for PC metastasis 56. Dense desmoplasia and extensive immunosuppression are two major factors that facilitate PC cell proliferation and evasion of immune surveillance. Hence, we investigated the immune landscape between SEMet^high^ and SEMet^low^ subtypes. According to two popular TIME signatures, we found that SEMet^low^ subtypes possessed a higher correlation with MDSCs and CAFs. MDSCs play an important role in the immunosuppression of PC, and PC could consistently induce the proliferation and mobility of MDSCs within the bone marrow to TIME 57. In the consistent, the contribution of CAFs to the biology of PC has generally been held to be tumor-promoting 58. Otherwise, several oncogenic biological processes were also enriched in SEMet^low^ subtypes, which suggested that the SEMet^low^ subtype was related to a malignant phenotype and therefore obtained a dismal prognosis in PC. Cancer immunotherapy implemented by ICBs has revolutionized the clinical management of solid tumors, including PC. Our results revealed that the expression of common ICBs (e.g, CD274 and PDCD1LG2) was upregulated in the SEMet^low^ subtype, implying that patients in the SEMet^low^ subtype may be more likely to benefit from ICBs therapy. The following TIDE algorithm and Submap analysis consistently confirmed the above-mentioned results. In general, our SEMet classifier is also a candidate biomarker for selecting PC patients who may be sensitive to immunotherapy.

Also, the genetic characteristics of the SEMet classifier were explored in our study. We found that the SEMet^low^ subtype tends to gain more TMB and higher mutation frequency of TP53, CDKN2A and KRAS. The mutations of all these three genes were widely studied and deemed as a contributor to the progression and metastasis of PC 59. The exploration of differences in CNA indicated that the amplification of 8q24.21, 8q24.22, 8q24.12 and deletion of 18q21.2, 9p21.3, 17p12 were significantly enriched in the SEMet^low^ subtype. 8q24.21 is reported to involve numerous cancer vulnerability loci and physically interact with oncogene MYC 60, 61. 18q21.2 harbors SMAD4, one of the most recurrently inactivated tumor suppressor genes in PC 62. Most notably, the deletion of 9p21.3 could promote metastasis by evading adaptive immunity in PC 63. All this evidence demonstrated that the genetic alteration between SEMet^high^ and SEMet^low^ subtypes, and the SEMet^low^ subtypes were significantly related to the proliferation and metastasis of PC.

For further exploring the underlying biological relationship between the SEMet^low^ subtypes and metastasis in PC, the difference in expression of marker genes related to biological functions and GSEA analysis was investigated. The results indicated that the SEMet^low^ subtype enriched in multiple metastasis-related pathways. Of which, metabolic reprogramming is an important component in the abnormal survival and growth of cancer cells 64, and the most common example is enhanced glycolysis. Glycolysis in PC cultures the vigorous growth of tumor cells by providing large amounts of substrates and promoting invasion and migration via the interaction of glycolytic enzymes and actin 65. ECM is known as a vital part composed of desmoplasia in PC. Continuous ECM remodeling and overexpression of matrix components promoted the recruitment of bone marrow cells, which are lately polarized to support tumor proliferation and invasion 66. Additionally, dysregulation of various tumor-related signaling pathways was found in the SEMet^low^ subtype. The predictive value of the SEMet classifier in metastatic PC was also validated in ICGC-AU-Array and GSE62452. Given the above, the SEMet^low^ subtype possessed metastatic features and therefore has been regarded as having “pro-metastasis” status in PC.

The prognostic value of 18 SEMet genes was also been investigated, and our SEMet classifier was demonstrated to the optimal model with outstanding prognostic performance. To be noted, we further observed that DKK1, TGM2 and E2F7 obtained significantly differential expression in PTs and HMs compared to Ns, and they have been reported to be related to the invasion and proliferation of PC 46, 47, 55. These SEMet genes were retrieved from HM-SE genes, while cancer cells have been shown to acquire SEs at oncogenes through the involvement of transcription factors (TFs). Interestingly, we demonstrated that E2F7 served as a TF and may mediate the transcriptional reprogramming of DKK1 and TGM2, which in turn contributes to the metastasis of PC.

As one of the main purposes of disease stratification, exploring tailored treatment strategies for a distinct subgroup is of great significance to maximize the therapeutic effect. Except for being informative regarding prognosis, the SEMet classifier could also be utilized for precise oncology as a potential biomarker to guide the treatment of metastatic PC. While a few patients benefit from targeted strategies and immunotherapy, gemcitabine remains the first-line drug for PC treatment. However, gemcitabine resistance is common and compromises long-term survival. After a comprehensive selection, FLM was screened out as the optimal compound which could enhance the efficacy of gemcitabine in metastatic PC. Until now, few research studies have concentrated on the therapeutic effect of FLM on cancer, especially PC 35. Our experiments demonstrated that FLM could significantly sensitize the metastatic PC to gemcitabine, highlighting the critical role of the SEMet classifier in drug screening.

SEMet classifier is superior to other methods in risk stratification and personalized treatment prediction as a classifier particularly developed for metastatic PC based on SE profiling. However, several limitations should be mentioned in our study. First, although the multi-center cohorts have been included in our study to construct and validate the SEMet classifier, the in-house data should be further added. Second, the deeper exploration of mechanisms in E2F7 targeting TGM2 and DKK1 was warranted. Finally, clinical validations of the SEMet classifier are necessary for promoting the translational value of our findings.

## Conclusion

In conclusion, based on HM-SE genes generated from H3K27ac ChIP-Seq data, the current study developed a consensus classifier (SEMet classifier) via 188 ML algorithm integrations in multicenter cohorts. This classifier not only exhibited robust predictive and prognostic performance but also stratified patients into distinct statuses at the immune and genomic levels. Among 18 SEMet genes in the SEMet classifier, DDK1 and TGM2 act in PC as a pro-metastasis factor targeted by E2F7. More importantly, we demonstrated that the SEMet^low^ subgroup was significantly correlated with more metastatic characteristics and further confirmed that FLM might significantly sensitize the metastatic PC to gemcitabine, throwing light on integrating tailored prognosis prediction with personalized treatment.

## Abbreviations

ATCC: American Type Culture Collection
AUC: area under the curve
CCLs: cancer cell lines
C-index: concordance index
CAN: copy number alteration
CPTAC: Clinical Proteomic Tumor Analysis Consortium
CRC: colorectal cancer
CTRP: Cancer Therapeutics Response Portal
DEGs: differentially expressed genes
Enet: elastic network
FLM: Flumethasone
FPKM: Fragments Per Kilobase of exon model per Million mapped fragments
GBM: generalized boosted regression modeling
GEO: Gene Expression Omnibus
GSVA: Gene set variation analysis
HCMDB: Human Cancer Metastasis Database
HMs: hepatic metastases
HNSCC: neck squamous cell carcinoma
ICBs: immune checkpoint blockages
ICGC: International Cancer Genome Consortium
IPS: immunophenoscore
k-NN: K-nearest neighbor
MDGs: methylation-driven genes
MDO: metastatic patient-derived organoids
ML: machine-learning
PC: pancreatic cancer
plsRcox: partial least squares regression for Cox
PTs: primary pancreatic tumors
RCC: renal cell carcinoma
ROC: receiver-operator characteristic
RSF: random survival forest
SE: super-enhancers
SEMet: super-enhancer-related metastatic
SuperPC: supervised principal components
survival-SVM: survival support vector machine
TCGA: The Cancer Genome Atlas
TFs: transcription factors
TIDE: The Tumor Immune Dysfunction and Exclusion
TMB: tumor mutation burden
