# Supplementary material for: Comprehensive machine learning-generated classifier identifies pro-metastatic characteristics and predicts individual treatment in pancreatic cancer: A multicenter cohort study based on super-enhancer profiling
Source: Theranostics. 2023 May 27;13(10):3290–309. doi: 10.7150/thno.84978 (PMC10283048; doi:10.7150/thno.84978)
Supplement: Supplementary file 1 — Supplementary methods, figures and tables. [file thnov13p3290s1.zip › s1/Supplementary Materials.pdf]

Supplementary Materials

- **Supplementary Methods**
- **Supplementary Figure S1**
- **Supplementary Figure S2**
- **Supplementary Figure S3**
- **Supplementary Figure S4**
- **Supplementary Figure S5**
- **Supplementary Figure S6**
- **Supplementary Figure S7**
- **Supplementary Figure S8**
- **Supplementary Figure S9**
- **Supplementary Figure S10**
- **Supplementary Figure S11**

Supplementary Methods

Univariate Cox regression analysis and bootstrapping method

First, preliminary screening was performed to include prognostic HM-SE genes in the training cohort (ICGC-AU-Array) via univariate Cox regression analysis, where genes with a p-value less than 0.01 were selected for further analysis. Next, the bootstrapping method was used to test the genes which passed initial filtering for robustness. We randomly extracted 70% of samples from the training cohort and performed univariate Cox regression analysis to assess the correlation between gene expression and prognosis. This procedure was iterated 1000 times and the genes incorporated in 90% of resample runs (achieved $p < 0.05$ in robustness testing) were identified as robust prognostic HM-SE genes.

Gene set enrichment analysis(GSEA)

Based on "c2.cp.kegg.v7.4.symbols" and "c5.bp.go.v7.4.symbols" gene sets, GSEA analysis was performed to explore the functional annotation of HM-SE genes using *clusterProfiler* package. A pathway with FDR $q < 0.25$ and $p < 0.05$ was defined as statistically significant.

Combination of integrative machine learning algorithms

To establish a comprehensive prognosis classifier in PC, we integrated 10 machine-learning algorithms and generated 188 combinations based on those prognostic HM-SE genes. Random survival forest (RSF) is a random forest method for the analysis of right-censored survival data [1]. RSF methodology extends Breiman's random forests (RF) method [2, 3]. In RF, randomization is introduced in two forms. First, a randomly drawn bootstrap sample of the data is used to grow a tree. Second, at each node of the tree, a randomly selected subset of variables (covariates) is chosen as candidate variables for splitting. Averaging over trees, in combination with the randomization used in growing a tree, enables RF to approximate rich classes of functions while maintaining low generalization errors. The RSF model was implemented via the randomForestSRC package [4]. The Enet, Lasso, and Ridge were implemented via the glmnet package [5]. The regularization parameter, λ, was determined by LOOCV, whereas the L1-L2 trade-off parameter, α, was set to 0-1 (interval =0.1). The stepwise Cox model was implemented via the survival package [6]. The direction mode of the stepwise search was set to "both", "backward", and "forward", respectively. The CoxBoost model was implemented via CoxBoost package [7], which is a Cox model by likelihood based boosting for a single survival endpoint or competing risks. For the CoxBoost model, we used LOOCV routine optimCoxBoostPenalty function to first determine the optimal penalty (amount of shrinkage). The plsRcox model was implemented via plsRcox package [8]. The SuperPC model was conducted via superpc package [9]. The GBM model was also implemented via gbm functions [10], which is used to fit the generalized boosted regression model. The survival-SVM model was utilized via survivalsvm package [11].

Published PC classifications prediction and comparison

The relationship between the SEMet classifier and reported PC molecular classifications was also explored. Five classical PC classifications have been analyzed, including Bailey's classification [12], Collisson's classification [13], Moffitt's tumor classification [14], Moffitt's stromal classification [14] and Puleo's classification [15]. Based on published signature genes and algorithms, unsupervised consensus clustering was applied for identifying the subtyping schemas of Bailey's classification, Collisson's classification, Moffitt's tumor classification and Moffitt's stromal classification on TCGA-PAAD dataset using the "ConsensusClusterPlus" package in R. For the prediction of Puleo's classification, we followed the pipeline defined by Puleo [15]. For each sample in the TCGA-PAAD dataset, the expression of genes in the centroids was selected, and Spearman rank correlation analysis was conducted between selected genes and 5 centroids. The subtype centroid with the highest correlation is the predicted class of the tested sample. The comparison between the distribution of five predicted classifications and our SEMet classifier was measured by Fisher's exact test. R package "ggalluvial" was utilized to plot the Sankey diagram. Cramer's V served as an effect size measurement for the association between the SEMet classifier and the other five classifications. It ranges from 0 to 1 where, 0 indicates no association between the two variables, and 1 indicates a perfect association between them.

RNA isolation and quantitative real-time PCR (qRT-PCR) assay

TRIzol reagent was used to extract total RNA, and AG Evo M-MLV RT Mix Kit was used to generate the corresponding cDNA. ABI 7500 Real-Time PCR machine was used for quantitative real-time PCR (Applied Biosystems, USA). The primers used are listed in Supplementary Table S3.

Invasion assay

To assess cell invasiveness, 24-well Transwell plates with 8μm pore polycarbonate membrane inserts (Corning) were employed. Ceturegel® Matrix at a concentration of 200 ng/mL was applied to the membrane (Yeason, 40187ES). Following 24 h of incubation, cells infiltrating onto the bottom surface of the membrane insert were fixed in 4% paraformaldehyde, stained with 0.5% crystal violet, and quantified by counting 3 random fields of view.

Western blotting

Western blotting was carried out as previously reported [16]. Cell lysates were separated using 10% sodium dodecyl sulfate-polyacrylamide gel electrophoresis (SDS-PAGE), and proteins were transferred to polyvinylidene difluoride (PVDF) membranes (Merck Millipore, USA). Te membranes were treated with primary antibodies overnight at 4 °C, followed by secondary antibody incubation. The Imaging System was used to detect target proteins (Tanon, China). Antibodies were used as listed:

β-Actin Rabbit mAb (1:5000, Abclonal, #AC048)

N-Cadherin Rabbit mAb (1:1000, Cell Signaling Technology, #13116)
E-Cadherin Rabbit pAb (1:1000, Proteintech, # 20874-1-AP)
Vimentin Rabbit mAb (1:1000, Cell Signaling Technology, #46173)
DKK1 Rabbit mAb (1:1000, Abcam, #ab109416)
Transglutaminase 2 Rabbit mAb (1:10000, Abcam, #ab109200)

Immunohistochemistry (IHC)

We firstly dewax and rehydrate the 3-m-thick paraffin-embedded sections of PDOX samples. Antigen retrieval was carried out in a pressure cooker for 15 minutes in 10 mM Tris with 1 mM EDTA (pH 9.0). The slices were then treated with specific antibodies against Ki-67 (1:200, Servicebio, #GB111499) overnight at 4 °C before being immunodetected with DAB the next day.

Animal studies

To demonstrate the function of E2F7 in metastasis of PC, luciferase-labeled Pan02 PC cells (D-Luciferin, Sodium Salt (Yeason, #40901)) with or without E2F7 knockdown were injected into the spleen vein of C57 mice, and further quantified by bioluminescence imaging (BLI). To evaluate the functions of flumethasone in vivo, metastatic lesion-derived organoids (2×10^6 crypts/0.1 mL PBS; n=5 in every group) were injected subcutaneously on the right fank of 4-week-old male BALB/c nude mice. Once the subcutaneous tumor volume reached 100 mm^2, the mice were randomly divided into four groups: saline, gemcitabine (6mg/kg, MedChemExpress #HY-B0003), flumethasone (0.1mg/kg, MedChemExpress #HY-B1051) [17] or combined administration (n=5/group). After four injections, the mice were killed on the third day after the last injection. The subcutaneous tumors were measured, fixed, and stained using IHC.

Screening of sensitive compounds targeted the SEMet^{low} PCs

The procedure of sensitive compounds selection was followed the protocal of Yang et al. [18]. The detailed data acquisition and analysis process are as follows:
(1) CTRP and PRISM database portals were used to download the gene expression matrix and drug sensitivity value (AUC) after drug intervention, and the cell lines with a missing value (NA value) more than 20% or from hematopoietic and lymphoid tissues were excluded. Finally, The information of 437 compounds in CTRP database and 1438 compounds in PRISM database were selected for subsequent analysis.
(2) The calcPhenotype() function in the “oncopredict” package [19] was used to predict the drug sensitivity of PC samples included in this study, and the predicted AUC value was obtained.
(3) Wilcoxon rank-sum test was used to analyze the differential drug sensitivity between the SEMet^{high} subgroup (top 20%) and the SEMet^{low} subgroup (bottom 20%). To identify compounds with higher

sensitivity (lower AUC values) in the the SEMet^{low} subgroup. The screening criteria were: log2FoldChange < -0.02 (CTRP)，log2FoldChange < -0.08 (PRISM);
(4) Spearman correlation analysis was performed between drug susceptibility values and SEMet scores to identify compounds with positive correlation coefficients. The screening criteria were: R > 0.2 (CTRP)，R > 0.3 (PRISM);
(5) By intersecting the compounds obtained in (3) and (4), several highly sensitive compounds could be obtained based on CTRP and PRISM databases, respectively.
For complimentary validation of the higher sensitivity drugs screened by the above methods, we also performed CMap analysis [20]. Firstly, the differences between paired PT samples and HM samples in GSE151580 database were analyzed, and the 300 genes with the most significant differences (including 150 significantly up-regulated genes and 150 significantly down-regulated genes) were screened out. Then, these differential genes were submitted to the CMap cloud platform (https://clue.io/query/) for analysis, and the CMap score of each compound was obtained. If the negative score of a compound is smaller, the gene expression pattern of the compound is opposite to the expression pattern of PC. It is suggested that this compound has a good therapeutic effect on PC.

Reference

1. Ishwaran H, Kogalur UB, Blackstone EH, Lauer MS. Random survival forests. 2008.
2. Breiman L. Random forests. Machine learning. 2001; 45: 5-32.
3. Breiman L. Bagging predictors. Machine learning. 1996; 24: 123-40.
4. Ishwaran H, Kogalur UB, Kogalur MUB. Package ‘randomForestSRC’. breast. 2023; 6.
5. Hastie T, Qian J. Glmnet vignette. Retrieved June. 2014; 9: 1-30.
6. Therneau TM, Lumley T. Package ‘survival’. R Top Doc. 2015; 128: 28-33.
7. Binder H, Binder MH. Package ‘CoxBoost’. Citeseer; 2015.
8. Bertrand F, Bastien P, Meyer N, Maumy-Bertrand M. plsRcox, Cox-Models in a high dimensional setting in R. Proceedings of User2014. 2014.
9. Wolfe MJ. Optimizing supercompilers for supercomputers: University of Illinois at Urbana-Champaign; 1982.
10. Ridgeway G. Generalized Boosted Models: A guide to the gbm package. Update. 2007; 1: 2007.
11. Van Belle V, Pelckmans K, Suykens JA, Van Huffel S. Survival SVM: a practical scalable algorithm. ESANN; 2008. p. 89-94.
12. Bailey P, Chang DK, Nones K, Johns AL, Patch AM, Gingras MC, et al. Genomic analyses identify molecular subtypes of pancreatic cancer. Nature. 2016; 531: 47-52.
13. Collisson EA, Sadanandam A, Olson P, Gibb WJ, Truitt M, Gu S, et al. Subtypes of pancreatic ductal adenocarcinoma and their differing responses to therapy. Nat Med. 2011; 17: 500-3.
14. Moffitt RA, Marayati R, Flate EL, Volmar KE, Loeza SG, Hoadley KA, et al. Virtual microdissection identifies distinct tumor- and stroma-specific subtypes of pancreatic ductal adenocarcinoma. Nat Genet. 2015; 47: 1168-78.
15. Puleo F, Nicolle R, Blum Y, Cros J, Marisa L, Demetter P, et al. Stratification of Pancreatic Ductal Adenocarcinomas Based on Tumor and Microenvironment Features. Gastroenterology. 2018; 155: 1999-2013.e3.

16. Lin J, Wang X, Zhai S, Shi M, Peng C, Deng X, et al. Hypoxia-induced exosomal circPDK1 promotes pancreatic cancer glycolysis via c-myc activation by modulating miR-628-3p/BPTF axis and degrading BIN1. J Hematol Oncol. 2022; 15: 128.
17. Zhou Y, Zhou Y, Wang K, Li T, Yang M, Wang R, et al. Flumethasone enhances the efficacy of chemotherapeutic drugs in lung cancer by inhibiting Nrf2 signaling pathway. Cancer Lett. 2020; 474: 94-105.
18. Yang C, Huang X, Li Y, Chen J, Lv Y, Dai S. Prognosis and personalized treatment prediction in TP53-mutant hepatocellular carcinoma: an in silico strategy towards precision oncology. Brief Bioinform. 2021; 22.
19. Maeser D, Gruener RF, Huang RS. oncoPredict: an R package for predicting in vivo or cancer patient drug response and biomarkers from cell line screening data. Briefings in bioinformatics. 2021; 22: bbab260.
20. Youens-Clark K, Faga B, Yap IV, Stein L, Ware D. CMap 1.01: a comparative mapping application for the Internet. Bioinformatics. 2009; 25: 3040-2.

Supplementary Figure S1

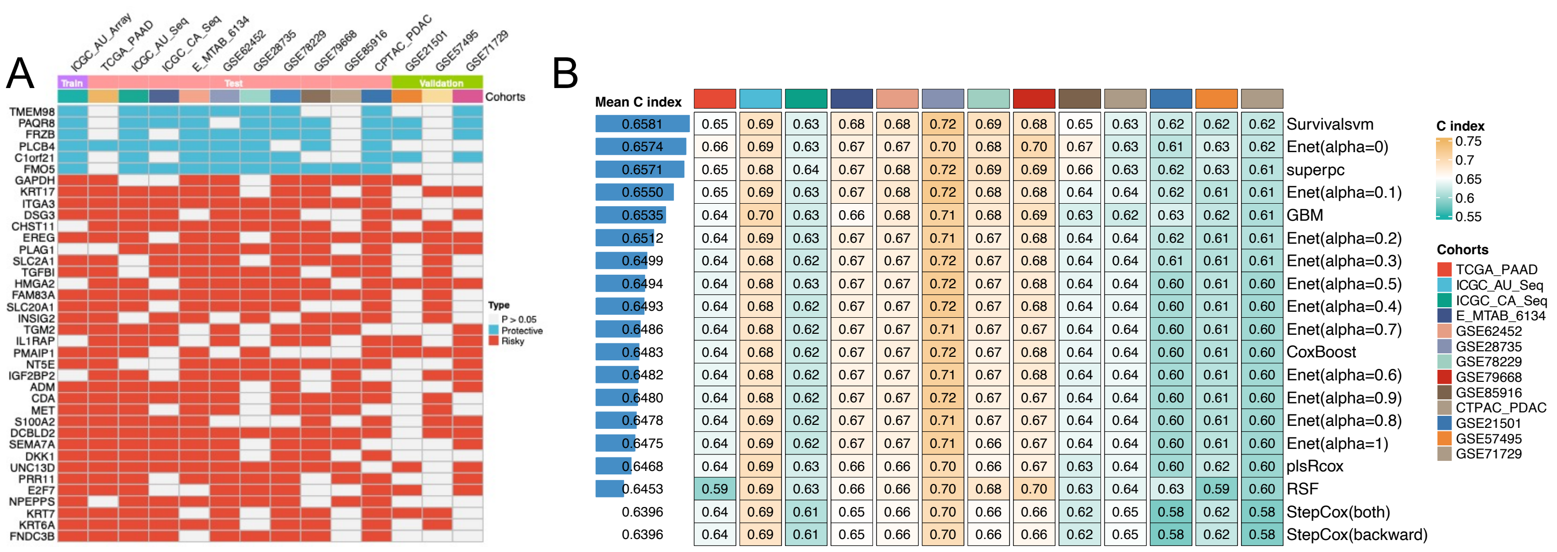

Supplementary Figure S1. Validation of SEMet classifier. (A) Univariate Cox analysis of 38 prognostic HM-SE genes was performed in all 14 enrolled cohorts. (B) The C-indexes of the SEMet classifier and models constructed by other methods for 18 SEMet genes in the 10 testing cohorts and 3 external validation cohorts.

Supplementary Figure S2

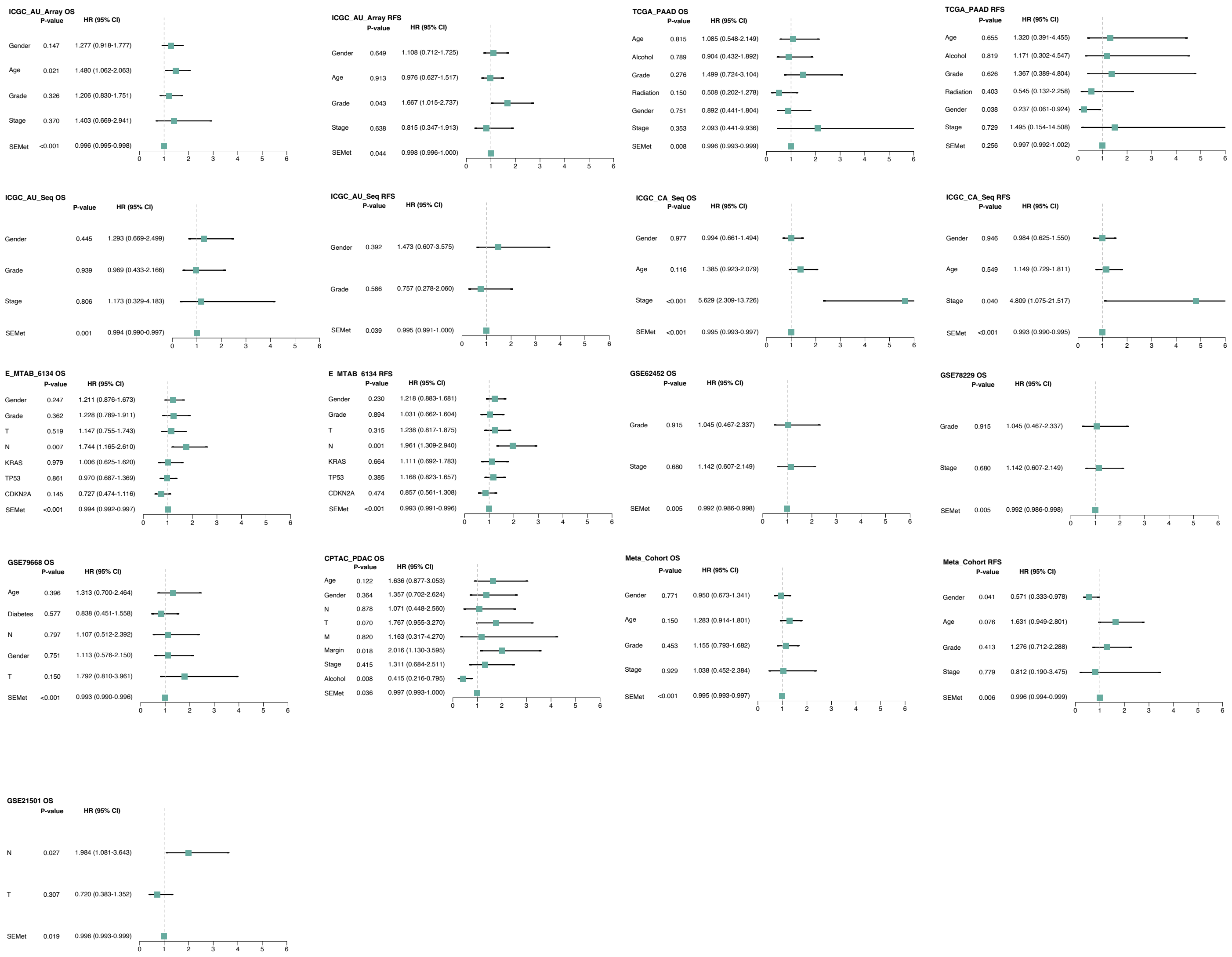

Supplementary Figure S2. Multivariate Cox regression analysis of OS and RFS in all enrolled cohorts.

Supplementary Figure S3

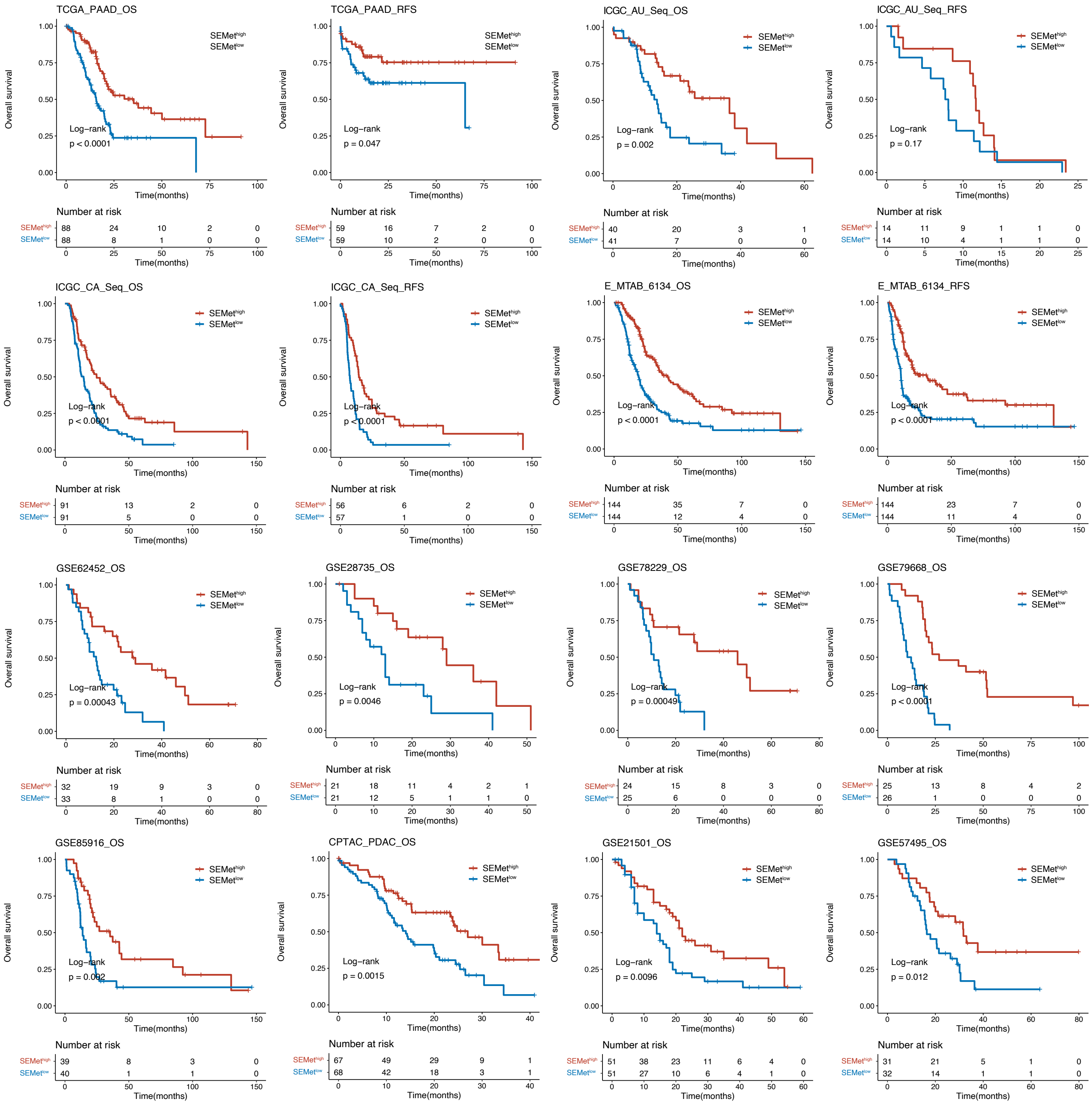

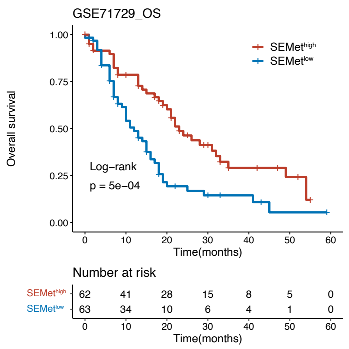

Supplementary Figure S3. Kaplan–Meier survival analysis for OS and RFS in 10 testing and 3 validation cohorts.

Supplementary Figure S4

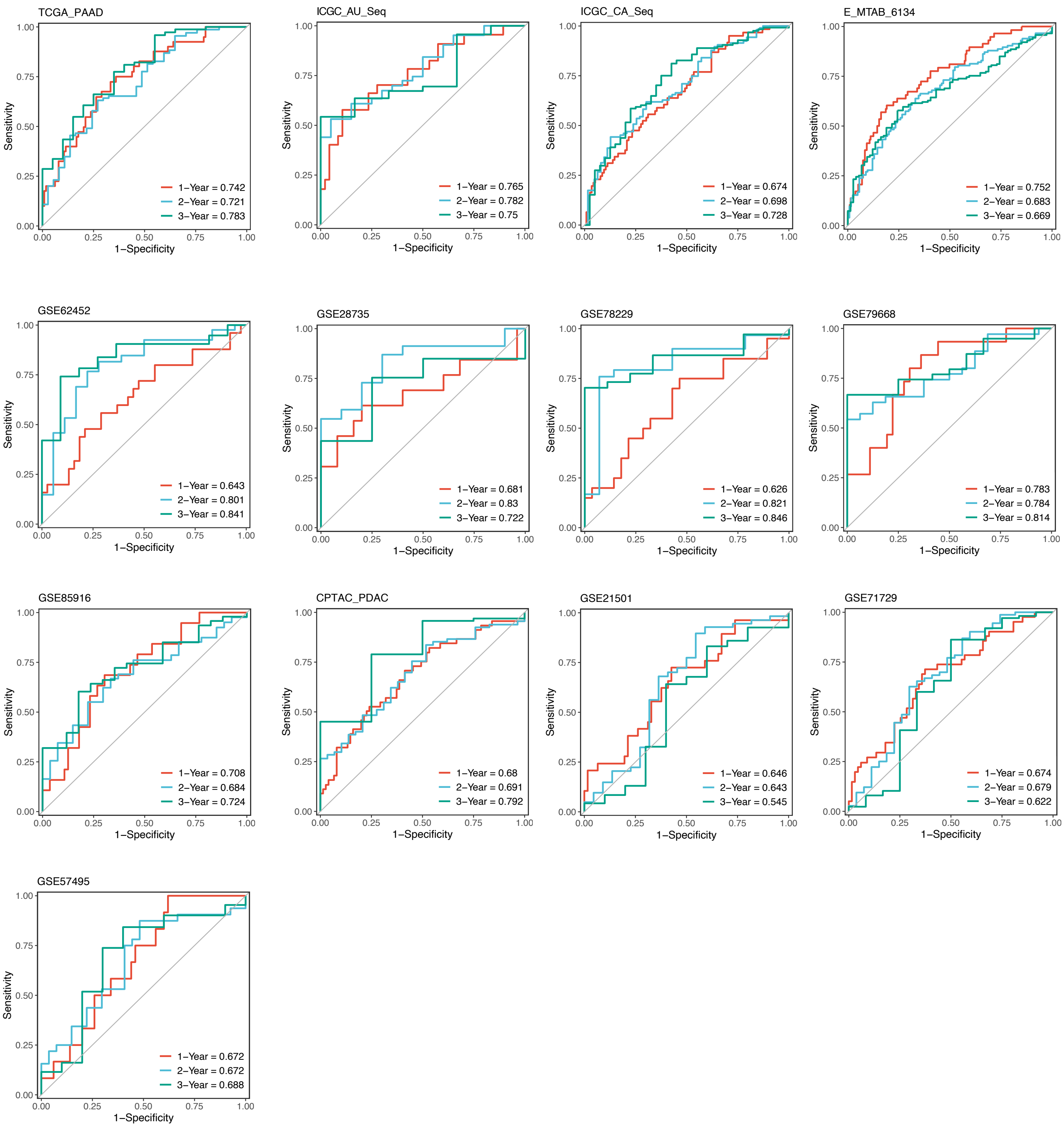

Supplementary Figure S4. Time-dependent receiver-operator characteristic (ROC) analysis for predicting 1-, 2-, and 3-year OS in 10 testing and 3 validation cohorts.

Supplementary Figure S5

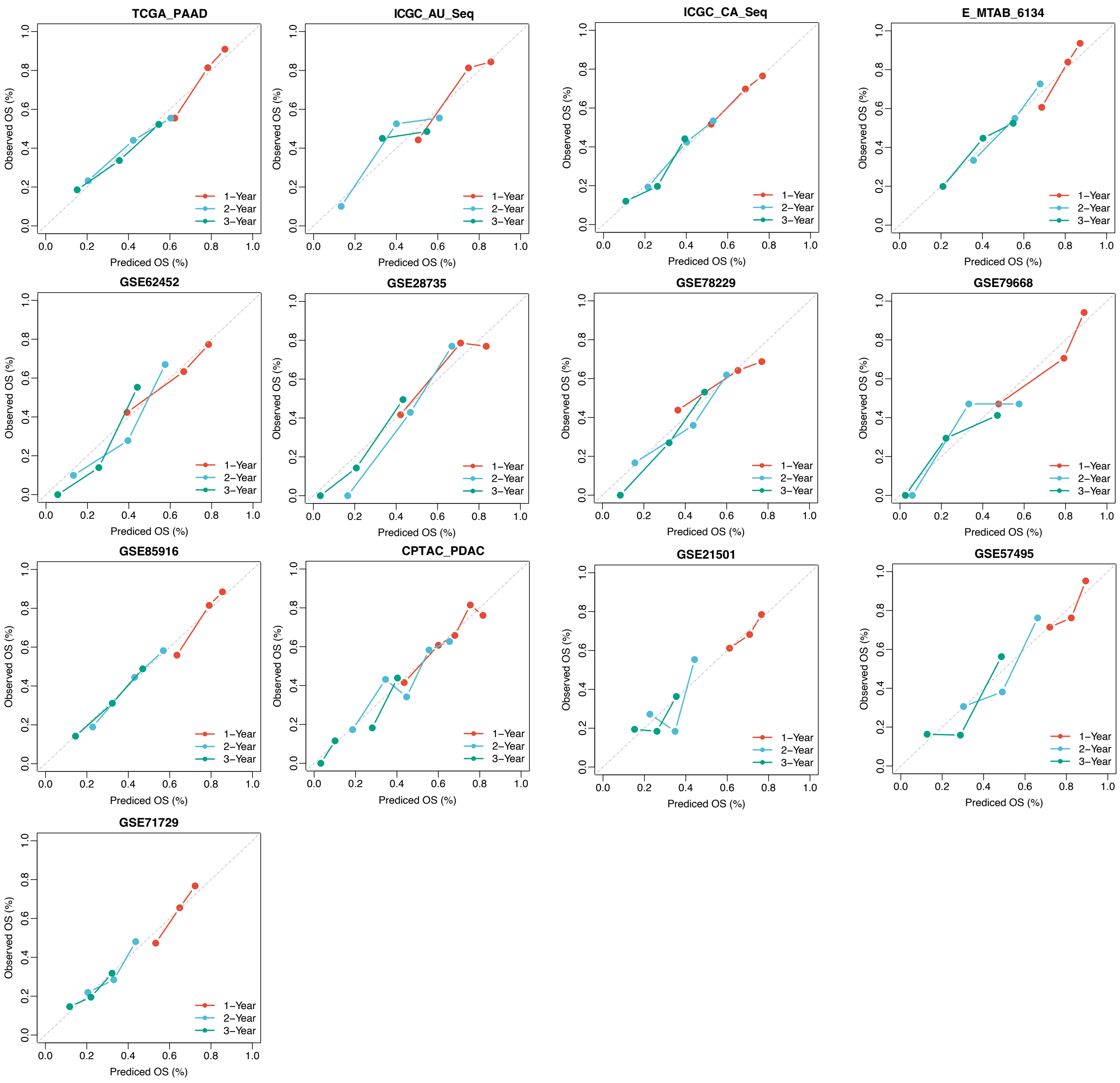

Supplementary Figure S5. Calibration curve for predicting 1-, 2-, and 3-year OS in 10 testing and 3 validation cohorts.

Supplementary Figure S6

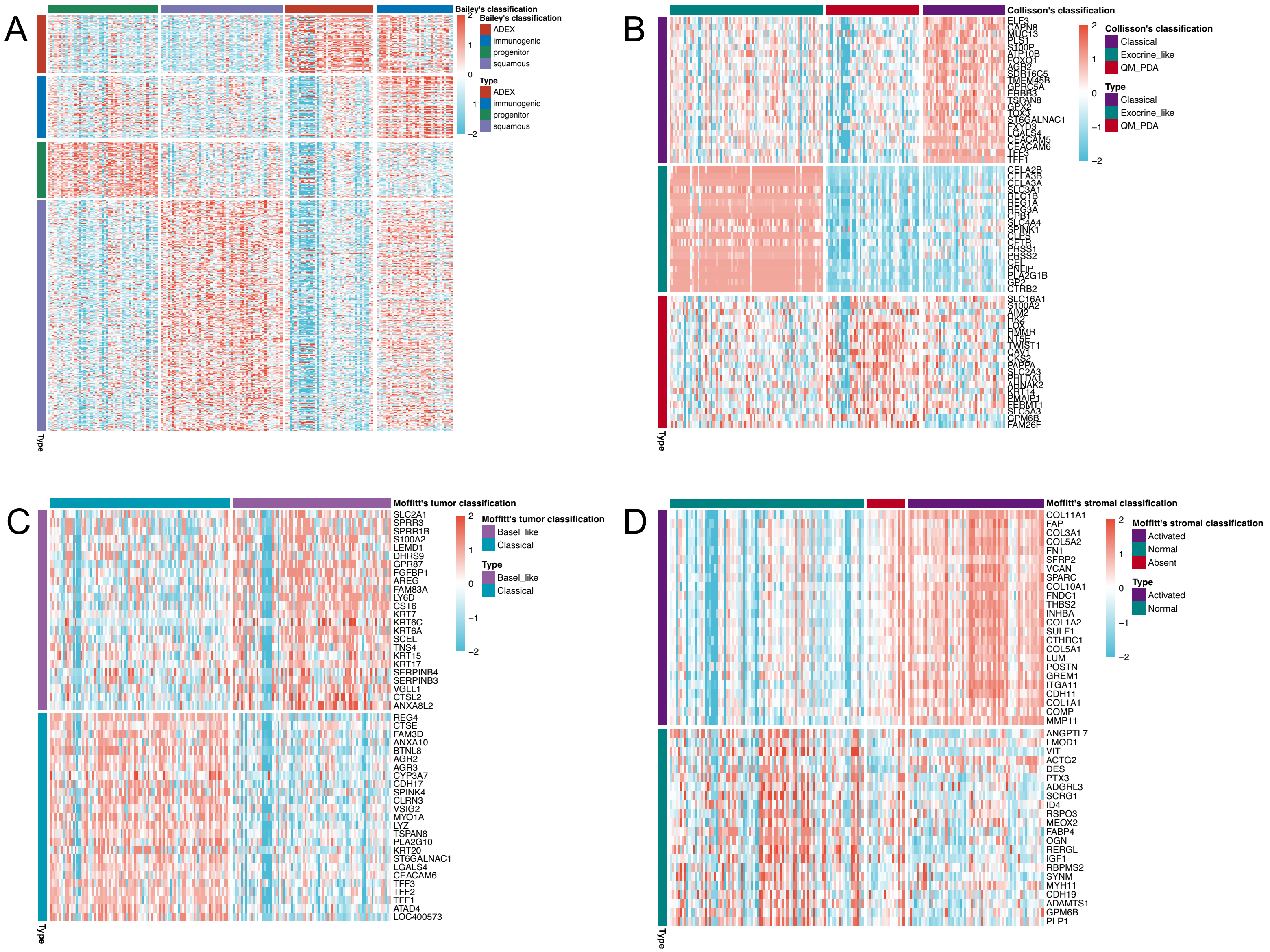

Supplementary Figure S6. Published PC subtypes prediction. Heatmap showed that we defined Bailey's classification (A), Collisson's classification (B), Moffitt's tumor classification (C), Moffitt's stromal classification (D) based on the published classifier exemplar genes.

Supplementary Figure S7

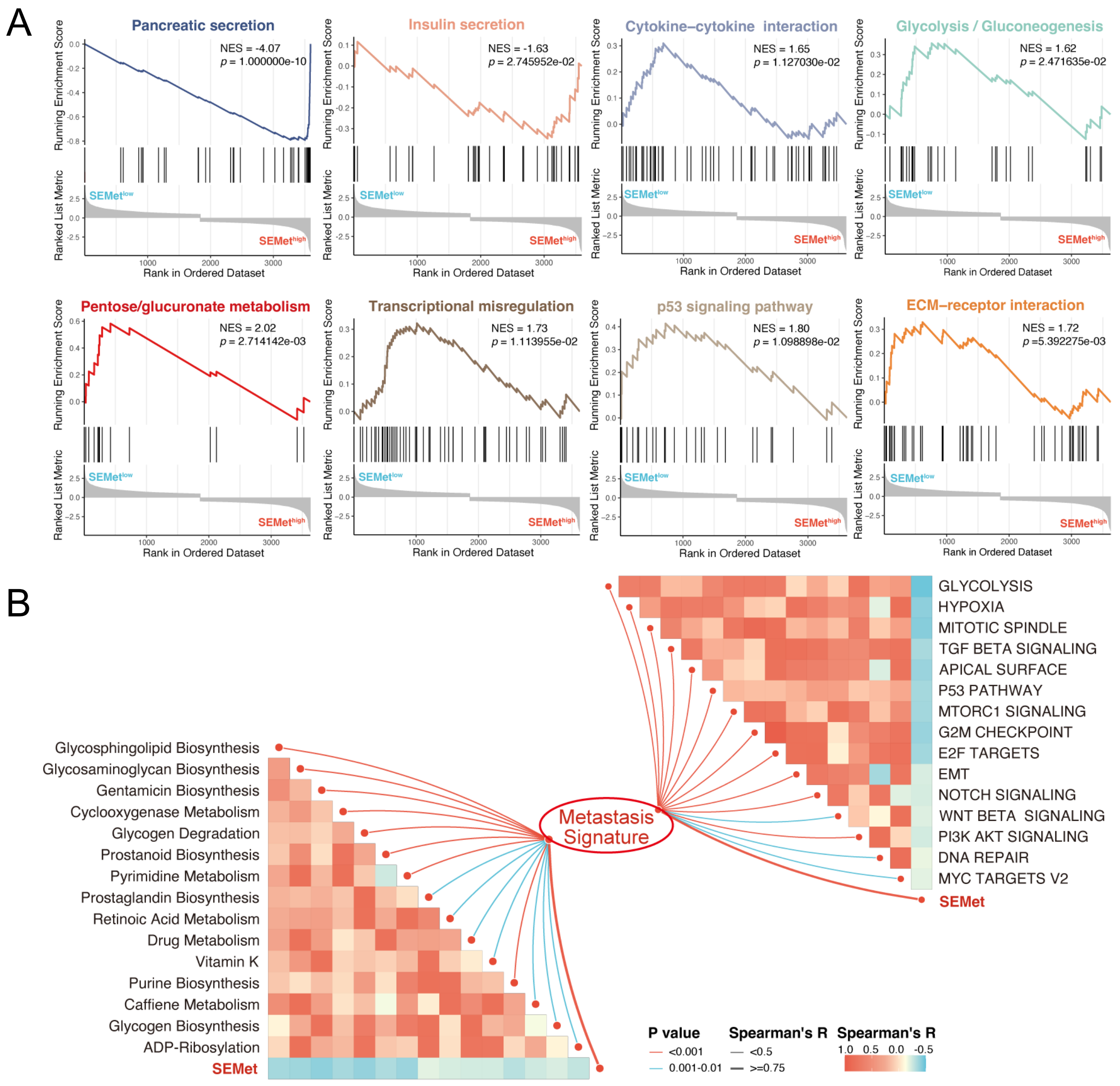

Supplementary Figure S7. Validation of the malignant phenotype in SEMet^{low} subgroups. (A) GSEA pathway analysis was conducted in SEMet^{high} and SEMet^{low} subgroups. (B) The metabolic and oncogenic pathways enriched in SEMet^{low} subgroups.

Supplementary Figure S8

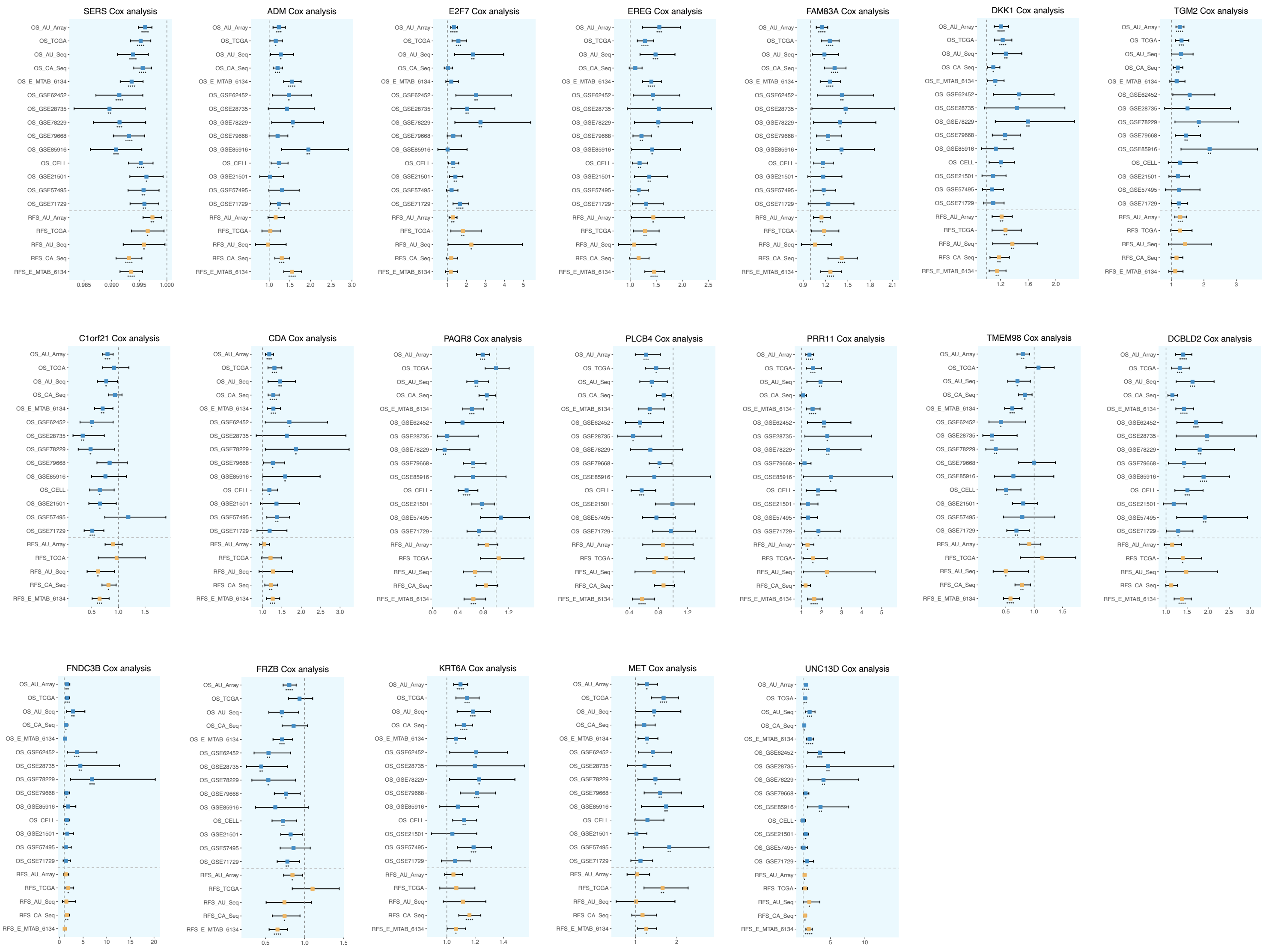

Supplementary Figure S8. Univariate Cox regression analysis of OS and DFS for 18 SEMet genes in 1 training set, 10 testing sets and 3 validation sets.

Supplementary Figure S9

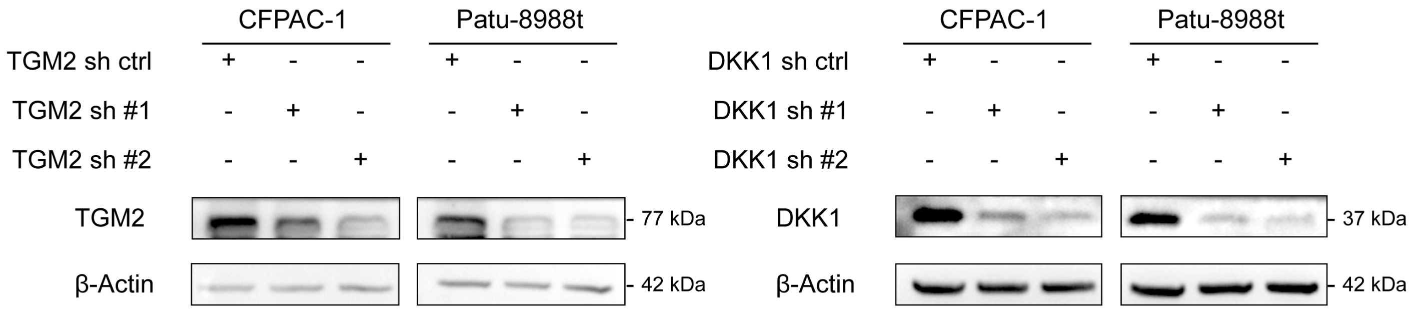

Supplementary Figure S9. Expression of TGM2 and DKK1 in sh-TGM2 and sh-DKK1.

Supplementary Figure S10

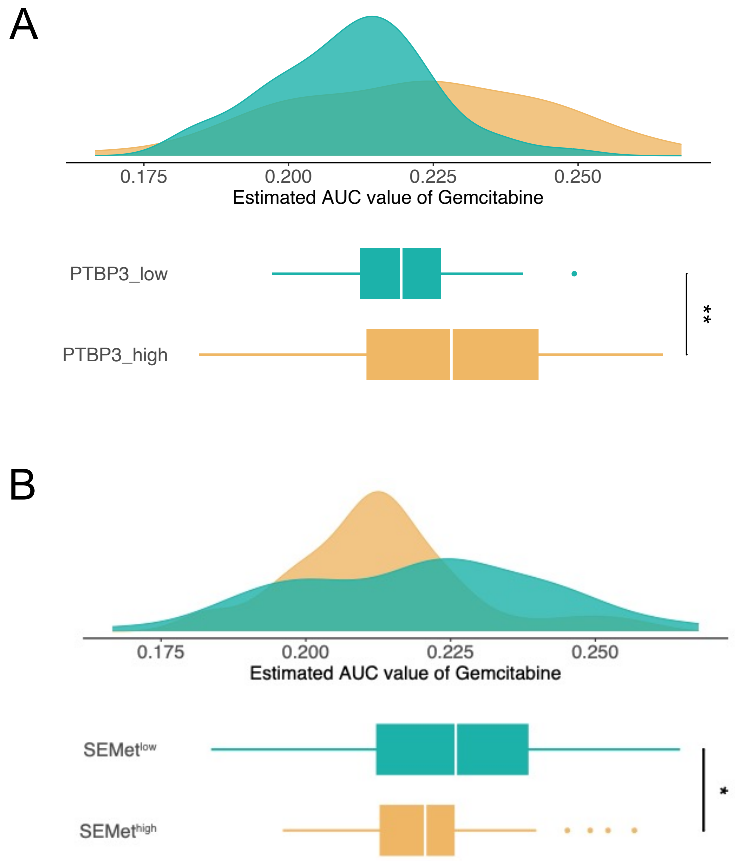

Supplementary Figure S10. Validation of the estimated AUC value in the TCGA-PAAD cohort. (A) Comparison of estimated gemcitabine's sensitivity between high and low PTBP3 expression groups. (B) Comparison of estimated gemcitabine's sensitivity between SEMet^{high} and SEMet^{low} groups.

Supplementary Figure S11

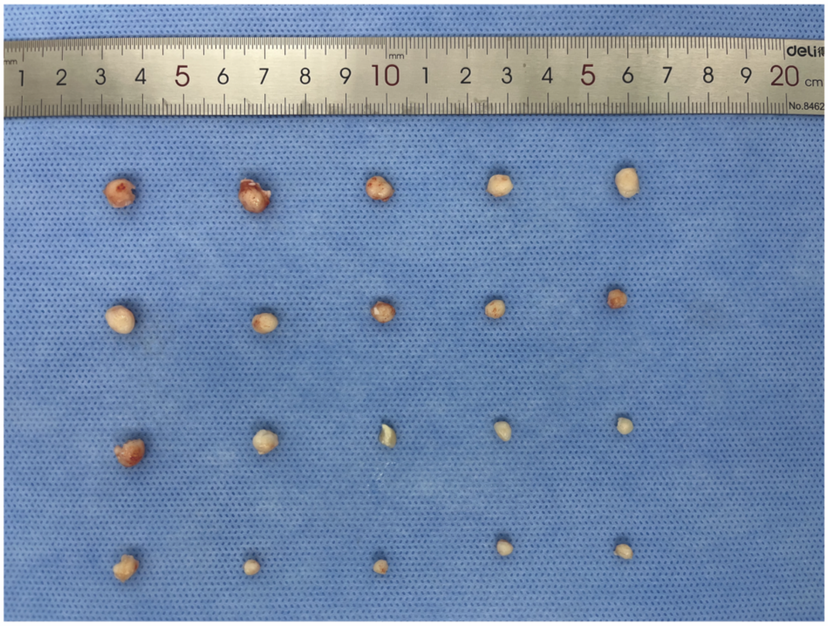

Supplementary Figure S11. The original figure of Figure 8G.
